# A heterodimeric SNX4­–SNX7 SNX-BAR autophagy complex coordinates ATG9A trafficking for efficient autophagosome assembly

**DOI:** 10.1242/jcs.246306

**Published:** 2020-07-15

**Authors:** Zuriñe Antón, Virginie M. S. Betin, Boris Simonetti, Colin J. Traer, Naomi Attar, Peter J. Cullen, Jon D. Lane

**Affiliations:** 1 Cell Biology Laboratories, School of Biochemistry, Medical Sciences Building, University of Bristol, Bristol BS8 1TD, UK; 2 Henry Wellcome Integrated Signalling Laboratories, School of Biochemistry, Medical Sciences Building, University of Bristol, Bristol BS8 1TD, UK

**Keywords:** Sorting nexin, SNX4, SNX7, SNX30, Endosomes, Autophagy, ATG9A

## Abstract

The sorting nexins (SNXs) are a family of peripheral membrane proteins that direct protein trafficking decisions within the endocytic network. Emerging evidence in yeast and mammalian cells implicates a subgroup of SNXs in selective and non-selective forms of autophagy. Using siRNA and CRISPR-Cas9, we demonstrate that the SNX-BAR protein SNX4 is needed for efficient LC3 (also known as MAP1LC3) lipidation and autophagosome assembly in mammalian cells. SNX-BARs exist as homo- and hetero-dimers, and we show that SNX4 forms functional heterodimers with either SNX7 or SNX30 that associate with tubulovesicular endocytic membranes. Detailed image-based analysis during the early stages of autophagosome assembly reveals that SNX4–SNX7 is an autophagy-specific SNX-BAR heterodimer, required for efficient recruitment and/or retention of core autophagy regulators at the nascent isolation membrane. SNX4 partially colocalises with juxtanuclear ATG9A-positive membranes, with our data linking the autophagy defect upon SNX4 disruption to the mis-trafficking and/or retention of ATG9A in the Golgi region. Taken together, our findings show that the SNX4–SNX7 heterodimer coordinates ATG9A trafficking within the endocytic network to establish productive autophagosome assembly sites, thus extending knowledge of SNXs as positive regulators of autophagy.

## Abstract (precis)

**Summary:** A heterodimeric SNX4–SNX7 SNX-BAR complex regulates mammalian autophagosome assembly through the control of ATG9 trafficking.

## INTRODUCTION

Macroautophagy (herein referred to as autophagy) describes the sequestration of cytoplasmic material within double membrane-bound vesicles (autophagosomes) that deliver their contents to lysosomes for degradation and recycling. Autophagosome assembly requires the concerted actions of conserved ATG proteins that are targeted to specialised endoplasmic reticulum (ER) subdomains (called omegasomes) that are enriched in the phosphoinositide, phosphatidylinositol 3-phosphate [PtdIns(3)P] (Axe et al., 2008). Consequently, molecules with affinity for either PtdIns(3)P and/or curved membrane profiles [for example, Bin/Amphiphysin/Rvs (BAR) domain-containing proteins (McMahon and Boucrot, 2015)] have been implicated in the control of autophagosome biogenesis. The WIPI family of PtdIns(3)P effector proteins are essential mediators of autophagosome assembly, coupling localised PtdIns(3)P to the recruitment of the ATG8 lipidation machinery [consisting of GABARAP and LC3 (also known as MAP1LC3) proteins] via direct binding (in the case of WIPI2b) to ATG16L1 (Dooley et al., 2015). Also acting during the autophagosome expansion phase is the BAR domain-containing protein, SH3GLB1 (also known as BIF-1 or Endophilin B1), which binds to UVRAG–Beclin-1 to stimulate the autophagy PIK3C3 kinase (also known as VPS34), whilst also facilitating trafficking of ATG9 to the autophagosome assembly site (Takahashi et al., 2007, 2011). Identification of further PtdIns(3)P effectors and/or BAR domain-containing proteins with the potential to influence autophagosome expansion and shaping remains a key objective.

Sorting nexins (SNXs) are a family of peripheral membrane proteins defined by the presence of a PX (phox homology) domain (Carlton et al., 2004; Cullen, 2008; Seet and Hong, 2006; Teasdale and Collins, 2012; Teasdale et al., 2001), and of the 33 SNXs annotated in the human genome, many interact with PtdIns(3)P. Because this lipid is enriched on early elements of the endocytic network (Gillooly et al., 2000), most SNXs are targeted to the cytosolic face of membrane-bound compartments that make up this diverse organelle. For one evolutionarily conserved subfamily of SNXs – the SNX-BARs (Carlton et al., 2004; Habermann, 2004) – the presence of an additional carboxy-terminal BAR domain conveys upon them the ability to generate and/or stabilise membrane tubules (Carlton et al., 2004). Mammalian cells possess twelve SNX-BAR family members – SNX1, SNX2, SNX4 through to SNX9, SNX18, SNX30, SNX32 and SNX33 (van Weering and Cullen, 2014; van Weering et al., 2010) – within which there is emerging evidence for a restricted pattern of BAR domain-mediated homo- and hetero-dimerisations (van Weering and Cullen, 2014; van Weering et al., 2010). Thus, the SH3 domain-containing SNX-BARs – SNX9, SNX18 and SNX33 – homodimerise to coordinate actin polymerisation with vesicle scission at sites of high membrane curvature (Dislich et al., 2011; Haberg et al., 2008; Lundmark and Carlsson, 2009; Schöneberg et al., 2017; van Weering et al., 2012) [although this conclusion remains controversial (Park et al., 2010)]. In contrast, SNX1, SNX2, SNX5 and SNX6 (and its neuronal counterpart SNX32) make up a membrane re-sculpturing coat complex named ESCPE-1, which consists of heterodimers of SNX1–SNX5, SNX1–SNX6, SNX2–SNX5 and SNX2–SNX6 (Simonetti et al., 2017, 2019).

Genetic screens in yeast have implicated the SNX-BARs, *SNX4* (also known as *ATG24* and *CVT13*) and *SNX42* (also known as *ATG20* and *CVT20*), as regulators of selective autophagy, including cytoplasm-to-vacuole targeting (Cvt) (Nice et al., 2002; reviewed in Lynch-Day and Klionsky, 2010), pexophagy (Ano et al., 2005; Deng et al., 2012), mitophagy (Kanki et al., 2009; Mendl et al., 2011), and selective degradation of fatty acid synthase (Shpilka et al., 2015). Snx4 colocalises with Atg8 at the phagophore assembly site (PAS) (Zhao et al., 2016), and perturbing the PtdIns(3)P-binding capabilities of these proteins prevents their association with the PAS, and impairs the Cvt pathway (Nice et al., 2002). Snx4 interacts with Atg17 (the yeast equivalent of mammalian RB1CC1, which is also known as FIP200) (Nice et al., 2002; Uetz et al., 2000), a protein that regulates Atg1-stimulated Atg9 trafficking to the PAS (Sekito et al., 2009). Taking this further, Popelka and colleagues argued that yeast Atg11 assembles with Snx4 and Snx42, replacing the non-selective autophagy Atg1 subcomplex (Atg17–Atg31–Atg29) to mediate selective autophagy (Popelka et al., 2017). Atg11 is a scaffolding protein that is specific for selective forms of autophagy in yeast (Zientara-Rytter and Subramani, 2019). In other hands, defects in non-selective autophagy have been reported for Snx4-null yeast (Ma et al., 2018); meanwhile, deletion of *SNX4* *or SNX42* in the background of other Golgi and/or endosomal mutants results in synthetic starvation-induced (non-selective) autophagy defects, suggesting compensatory masking of phenotypes in single deletion settings (Ohashi and Munro, 2010). In yeast, a series of dimeric interactions defined by weak Snx4–Snx4 homodimers and more pronounced Snx4–Snx41 and Snx4–Snx42 heterodimers have been described (Hettema et al., 2003; Ito et al., 2001; Popelka et al., 2017; Uetz et al., 2000; Vollert and Uetz, 2004), and these findings are consistent with data obtained using recombinant human proteins (Traer et al., 2007). Which of mammalian SNX7 and SNX30 is the functional homologue of yeast Snx41 and Snx42 is difficult to establish given their respective sequence similarities, and precise roles for homo- or hetero-dimeric complexes established within this group of proteins remain uncertain. Phylogeny and dimerisation patterns suggest that Snx42 is likely to be the yeast equivalent of mammalian SNX30 (Popelka et al., 2017), and intriguingly, an indirect role for Snx4–Snx42 during autophagosome-to-vacuolar fusion via coordinated mobilisation of phosphatidylserine-containing membranes from the endocytic compartment has been described (Ma et al., 2018).

An imaging-based LC3 lipidation screen has described a role for an SH3-containing SNX-BAR, SNX18, during autophagy in mammalian cells (Knævelsrud et al., 2013). SNX18 contains a conserved LC3-interacting (LIR) motif, and binds dynamin-2 independently of the LIR to mediate ATG9A trafficking from the recycling endosome and ATG16L1- and LC3-positive membrane delivery to the autophagosome assembly site (Knævelsrud et al., 2013; Søreng et al., 2018). Here, we have tested whether SNX4 also contributes to autophagy. Furthermore, we have investigated the concept of restricted patterns of dimeric interactions within the mammalian SNX-BAR family, asking how this behaviour modulates the autophagy response with respect to SNX4. We present data establishing SNX4 as a core component of two heterodimeric endosomal-associated complexes described by SNX4–SNX7 and SNX4–SNX30. Moreover, we show that the SNX4–SNX7 heterodimer is a positive regulator of autophagosome assembly in mammalian cells. Our data suggest that SNX4 complexes promote autophagosome assembly kinetics by mobilising ATG9A-associated membranes from the juxtanuclear area of the cell in response to autophagy stimulus.

## RESULTS

### siRNA suppression of SNX4 expression impairs autophagy

**Fig. 1.**
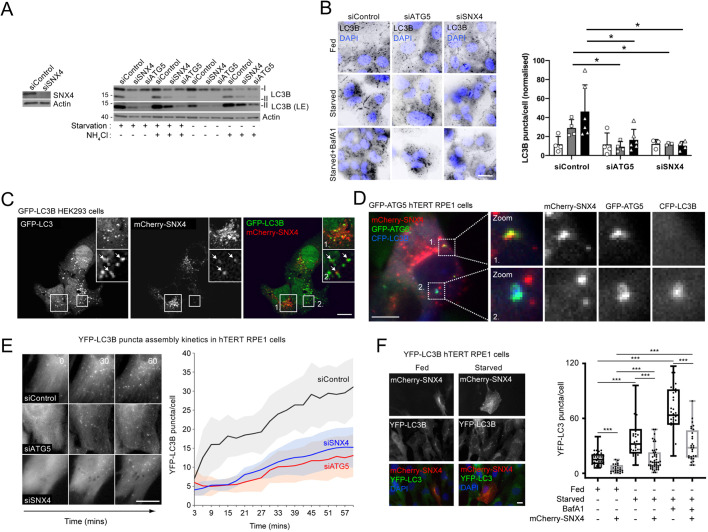
**SNX4 is a positive regulator of mammalian autophagy.** (A) Immunoblotting of lysates of hTERT-RPE1 cells treated with siRNAs targeting SNX4, ATG5, or with a non-targeting siControl. For these experiments, hTERT-RPE1 cells were incubated for 1 h in serum and amino acid free medium (starvation) in the absence or presence of 50 mM NH_4_Cl. LE=long exposure. Actin is shown as a loading control. Size markers indicated are in kDa. (B) Endogenous LC3B puncta quantitation in hTERT-RPE1 cells treated with siRNAs targeting SNX4, ATG5, or with a non-targeting siControl, in full nutrients (fed) and after starvation (1 h)±BafA1. Example images to the left; quantitation to the right. Mean±s.d. of ≥3 experiments. *P<0.05. (C) Confocal images of GFP–LC3B HEK293 cells transiently expressing mCherry–SNX4 under starvation conditions. Coincident staining can be seen between some punctate structures (indicated by arrows). Boxes indicate regions shown at higher magnification in inset images. (D) Wide-field live-cell imaging of GFP–ATG5 hTERT RPE1 cells transiently co-expressing mCherry–SNX4 and CFP–LC3B. Examples of apparent colocalisation between SNX4 and ATG5/LC3B in the regions indicated by dashed boxes can be seen in the magnified images (zoom). (E) YFP–LC3B puncta assembly kinetics during starvation in hTERT RPE1 cells treated with siControl, siSNX4 or siATG5. Example fields to the left; quantitation to the right. Mean±s.e.m. (F) Autophagy response (LC3B puncta) in YFP–LC3B hTERT RPE1 cells transiently expressing mCherry–SNX4 in fed or starved conditions. Example fields to the left; quantitation to the right. Boxes indicate the interquartile range (IQR) and the horizontal bar marks the median. Whiskers indicate minimum and maximum values. ***P<0.001. Scale bars: 10 µm.

Given the evidence implicating Snx4 in various forms of autophagy in yeast, we tested for possible roles for mammalian SNX4 during amino acid and growth factor starvation-induced autophagy in cell culture by treating hTERT-immortalised retinal pigment epithelial (hTERT-RPE1) with siRNAs targeting SNX4. Immunoblotting-based analysis of autophagic LC3B (MAP1LC3B) lipidation during starvation revealed impaired conversion to lipid-conjugated LC3B-II (Fig. 1A), and significantly fewer autophagosomes in hTERT-RPE1 cells labelled with anti-LC3B antibodies (reduced to a level similar to that observed after ATG5 silencing) (Fig. 1B). This effect was also seen with an additional siSNX4 oligonucleotide (Fig. S1A), and in a different cell line, GFP–LC3B-expressing HEK293 cells (Köchl et al., 2006) (Fig. S1B).

We have previously shown that SNX4 colocalises with peripheral endocytic membranes and the juxtanuclear RAB11-positive endocytic recycling compartment in full nutrient conditions (Traer et al., 2007). As a BAR domain protein with affinity for PtdIns(3)P, we explored the possibility that SNX4 influences autophagy via its association with autophagosomes and/or PtdIns(3)P-enriched autophagosome assembly sites. Transient expression of mCherry–SNX4 in stable GFP–LC3B HEK293 cells revealed that whilst the majority of SNX4-positive membrane structures were spatially separated from GFP–LC3B puncta, a minor fraction could be found in close proximity to GFP–LC3B-labelled autophagosomes (Fig. 1C). Similarly, in hTERT-RPE1 cells stably expressing GFP–ATG5 (MacVicar et al., 2015) and transiently expressing mCherry–SNX4 and CFP–LC3B, we recorded incidences of SNX4-positive structures juxtaposed with GFP–ATG5-positive autophagosome assembly sites, and further examples of mCherry–SNX4 structures in close proximity to GFP–ATG5- and CFP–LC3B-positive structures that were likely to be forming phagophores (Fig. 1D), suggesting that any interaction is likely to occur during autophagosome assembly, at around the ATG8 lipidation stage.

To begin to understand how SNX4 influences autophagy, we imaged autophagosome assembly kinetics during amino acid and growth factor starvation in hTERT-RPE1 cells stably expressing YFP–LC3B (Fig. 1E). We compared control, *ATG5*, and *SNX4* siRNA silenced cells, assessing cumulative YFP–LC3B puncta numbers without inclusion of lysosomal blocking reagents. The kinetics of YFP–LC3B puncta assembly were clearly altered when *SNX4* was silenced, with puncta formation rates decreased to a level that was comparable with *ATG5*-silenced cells (Fig. 1E). This suggested that the reduction in stimulated LC3B-positive autophagosome numbers observed at steady state in fixed cells (Fig. 1B) was unlikely to be due to increased LC3B turnover due to enhanced autophagic flux. Because overexpression of SNX18 enhances LC3 lipidation without influencing autophagic flux (Knævelsrud et al., 2013), we tested the effects of mCherry–SNX4 overexpression on the autophagy response in hTERT-RPE1 cells stably expressing YFP–LC3B, counting basal and starvation-induced LC3B puncta in mCherry–SNX4-positive cells (Fig. 1F). Basal (fed state) LC3B numbers were significantly lower in mCherry–SNX4 overexpressing cells when compared with neighbouring, untransfected cells, and this pattern was repeated following starvation in the absence or presence of the vacuolar H^+^-ATPase inhibitor, Bafilomycin A1 (BafA1) (Fig. 1F). This suggests that SNX4 overexpression impairs autophagosome assembly, rather than increasing the rate of lysosomal flux. Steady state numbers of GFP–ATG5-positive assembly sites were not affected by SNX4 overexpression (Fig. S2). These data suggest that the balance of expression of different autophagy-influencing SNX-BARs effects autophagy in different ways; whereas SNX18 overexpression enhances LC3 lipidation (Knævelsrud et al., 2013), elevated SNX4 expression clearly suppressed the starvation-mediated autophagy response at the level of LC3B puncta formation.

The observed differences in autophagy responses in cells overexpressing SNX4 might relate to changes in the structure or function of the endocytic compartment. To examine this, we generated hTERT-RPE1 cell lines stably overexpressing GFP–SNX4. In these cells, the number of distinct EEA1-positive early endocytic structures was significantly lower than seen in control, GFP-expressing hTERT-RPE1 cells under both fed and starvation conditions (Fig. S3A). Analysis of CD63-positive late endosomes and lysosomes revealed that these were significantly more abundant in fed GFP–SNX4 expressing hTERT-RPE1 cells, but that there was a clear absence of induced increases in numbers of CD63 puncta following amino acid and growth factor starvation when compared to control GFP-expressing cells (Fig. S3B). This suggests that SNX4 overexpression upsets the balance of membrane trafficking within the endolysosomal compartment. To rule out the possibility that SNX4 overexpression disturbs endocytic organelle properties and autophagosome assembly kinetics by competing for and/or blocking available PtdIns(3)P on endosomes and autophagosome assembly sites, we analysed LC3B puncta numbers in cells overexpressing a similar PtdIns(3)P-binding motif, mCherry–2×FYVE (Fig. S4). No differences in LC3B puncta numbers were recorded in mCherry–2×FYVE expressing cells in fed or starved conditions or when treated with the mTORC1/2 inhibitor AZD8055 (AZD), when compared to untransfected controls (Fig. S4). Taken together, these data suggest that reduced autophagosome assembly and LC3B lipidation kinetics caused by changes in the levels of cytosolic SNX4 in nutrient-starved cells (by overexpression or by siRNA silencing) might be linked to altered endomembrane properties. This prompted us to further explore the endosomal biology of SNX4 and its relationship with the autophagy regulatory system.

### SNX4 displays a restricted pattern of interactions with two other SNX-BAR family members, SNX7 and SNX30

**Fig. 2.**
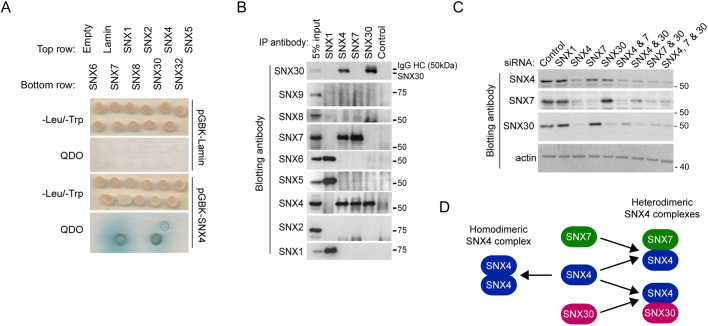
**SNX4 forms the core component of two distinct SNX-BAR heterodimers.** (A) Directed yeast two-hybrid test for SNX-BAR interactions. Lamin is included as a negative control. QDO, quadruple drop-out medium. (B) Native immunoprecipitations of SNX1, SNX4, SNX7 and SNX30 in HeLa cells. Immunoprecipitates were blotted for the SNXs shown. Size markers indicated are in kDa. (C) siRNA silencing of SNX1 and SNX4, SNX7, SNX30 alone and in combination in HeLa cells. Lysates were immunoblotted using the antibodies shown. (D) Schematic of the likely dimerisations that exist between SNX4, SNX7 and SNX30 SNX-BAR pairings.

Within the SNX-BAR family, there is strong evidence for a restricted pattern of BAR domain-mediated dimerisations, leading to the formation of specific SNX-BAR homo- and hetero-dimers (Cullen, 2008; Simonetti et al., 2017; van Weering et al., 2012, 2010). Previous analysis using overexpressed proteins indicated that SNX4 forms weak homodimers and relatively stable heterodimers with the SNX-BARs SNX7 and SNX30 (van Weering et al., 2012). To begin to assess whether SNX4 influences autophagy in homodimeric form or in a heterodimeric complex with another SNX-BAR, we carried out directed yeast two-hybrid screens, probing the interactions of full-length SNX4 with other SNX-BARs (SNX7, SNX8 and SNX30, and the ESCPE-1 SNX-BARs SNX1, SNX2, SNX5, SNX6 and SNX32) (Fig. 2A). SNX4 did not form any detectable associations with ESCPE-1 SNX-BARs or with SNX8; however, a weak interaction with itself and strong interactions with both SNX7 and SNX30 were observed (Fig. 2A), consistent with previous *in vitro* pulldown analysis (van Weering et al., 2012). A limitation of interaction studies requiring the overexpression of one or more putative partner proteins concerns the forcing of interactions that might not be physiologically relevant *in vivo*. We therefore sought further information on the specificity of interactions between SNX4, SNX7 and SNX30 through immunoprecipitation of endogenous proteins (Fig. 2B). Confirming previously published data describing the restricted pattern of heterodimeric interactions between SNX1–SNX5 and SNX1–SNX6 (Wassmer et al., 2007, 2009), immunoprecipitates of SNX1 were positive for endogenous SNX5 and SNX6 (Fig. 2B). Within these immunoprecipitates, we failed to detect endogenous SNX30, SNX9, SNX8, SNX7 or SNX4 (Fig. 2B). Conversely, immunoprecipitates of endogenous SNX4 were characterised by the presence of endogenous SNX7 and SNX30, and the lack of any detectable retromer SNX-BARs, or SNX9 and SNX8 (Fig. 2B). More specifically, within immunoprecipitates of endogenous SNX7 and SNX30, the only other sorting nexin detected in each case was SNX4 (Fig. 2B). Overall, these data clearly establish that, at the level of endogenous protein expression, SNX4 forms the core of two distinct heterodimeric complexes; SNX4–SNX7 and SNX4–SNX30.

For the mammalian ESCPE-1 SNX-BARs, it is well documented that siRNA suppression of the expression of SNX1 and SNX2 destablises SNX5 and SNX6, leading to their degradation (Carlton et al., 2004, 2005; Simonetti et al., 2017; Wassmer et al., 2007). Because this could have consequences for our interpretation of autophagy in *SNX4*-silenced cells, we considered whether a similar scenario exists for the SNX4–SNX7 and SNX4–SNX30 dimers: if SNX4 is the core component, does its suppression also lead to a loss of SNX7 and/or SNX30? Indeed, siRNA suppression of SNX4 protein expression caused a clear decrease in the levels of both SNX7 and SNX30 (Fig. 2C). In contrast, suppression of the retromer component SNX1 had no discernible effect on SNX4, SNX7 or SNX30 (Fig. 2C). Interestingly, effective silencing of SNX7 expression resulted in a small but clearly detectable drop in SNX4 protein levels, while the expression of SNX30 appeared unaffected (Fig. 2C). Likewise, strong suppression of SNX30 expression again induced a detectable decrease in SNX4, but this had no effect on SNX7 expression (Fig. 2C). Taken together, these data are consistent with SNX4 forming the core of two distinct SNX4–SNX7 and SNX4–SNX30 complexes (Fig. 2D). Although changes at the transcriptional level cannot be excluded, we propose that, upon SNX4 suppression, the loss of the core component destabilises the other constituents, whereas upon suppression of an individual complex-specific component, such as SNX7, the presence of the core SNX4 component allows the stabilisation of the SNX4–SNX30 complex (and vice versa under conditions of SNX30 suppression). Indeed, as one would predict from such a model, dual suppression of SNX7 together with SNX30 led to a pronounced loss in the levels of SNX4 (Fig. 2C).

### SNX4 colocalises with SNX7 and SNX30 predominantly on early endosomes

**Fig. 3.**
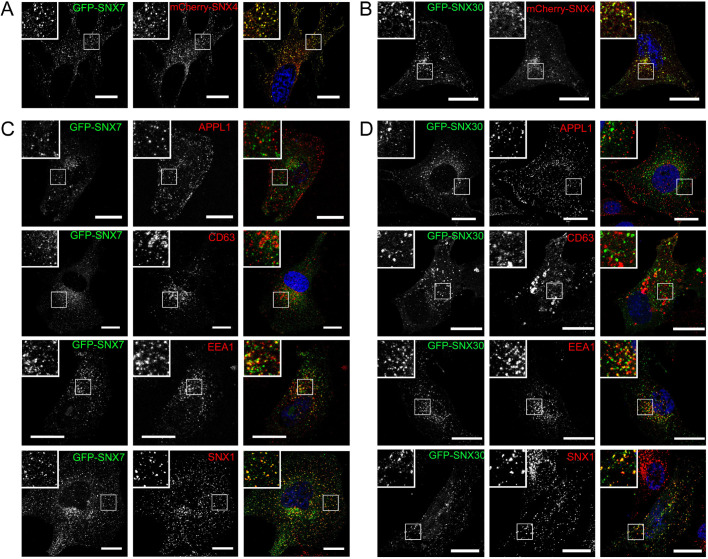
**Subcellular localisation of SNX4, SNX7 and SNX30.** (A,B) Confocal images of HeLa cells expressing GFP–SNX7 (A) or GFP–SNX30 (B) with mCherry–SNX4. The requirement for SNX4 co-expression is further assessed in Fig. S5. (C,D) Analysis of GFP–SNX7 (C) and GFP–SNX30 (D) endosomal targeting in HeLa cells co-expressing FLAG–SNX4 (not shown) and counterstained for various endocytic markers (APPL1, CD63, EEA1 and SNX1). For A–D, merge images with DAPI staining are shown on the right. Boxes indicate regions shown at higher magnification in inset images. Scale bars: 10 µm.

Previous studies have established that SNX4 is targeted to early endosomes via association of its PX domain with PtdIns(3)P (Leprince et al., 2003; Skånland et al., 2009, 2007; Teasdale et al., 2001; Traer et al., 2007). Although the phosphoinositide-binding characteristics of SNX30 have yet to be described, SNX7 has been shown to associate specifically with PtdIns(3)P (Xu et al., 2001). To examine whether SNX7 and SNX30 also associate with PtdIns(3)P-enriched early endosomes, we used lentiviruses to express GFP- or mCherry-tagged full-length human SNX7 or SNX30 at a level that did not cause noticeable remodelling of endosomal membranes (Carlton et al., 2004; Cozier et al., 2002). Unlike the situation when expressing GFP-tagged SNX4 (Traer et al., 2007), lentiviral transduction of HeLa cells with lentiviruses encoding GFP–SNX7 or GFP–SNX30 alone resulted in relatively weak levels of expression, and in those cells where a signal could be observed, both gave predominantly cytosolic staining patterns with some evidence of punctate staining (Fig. S5). Interestingly, when these same viruses were used to co-transduce HeLa and hTERT-RPE1 cells alongside a virus encoding mCherry–SNX4, clear colocalisation of mCherry–SNX4 and GFP–SNX7 or GFP–SNX30 was readily observed on puncta that were dispersed throughout the cytoplasm (Fig. 3A,B; Fig. S5A,B). Based on the biochemical evidence that SNX7 and SNX30 are unable to establish stable homodimers (Fig. 2), and given that dimerisation is a prerequisite for assembly of a functional membrane binding BAR domain (Peter et al., 2004), we interpret these data to mean that in the absence of co-expression with SNX4, SNX7 and SNX30 exist as unstable monomers that have insufficient affinity for PtdIns(3)P to attain steady-state endosomal association. Indeed, the importance of the combined, coincident membrane-binding activities of the PX and BAR domains is well established in this context (Carlton et al., 2004; Traer et al., 2007). Although the colocalisation of SNX7 and SNX30 with SNX4 was consistent with an association with early endosomes, we addressed this directly by co-transducing HeLa cells with lentiviruses encoding GFP–SNX7 or GFP–SNX30 together with a lentivirus encoding for FLAG-tagged SNX4, and counter stained with a variety of early and late endosomal markers (Fig. 3C,D). Confocal imaging revealed that in the presence of FLAG–SNX4, both GFP–SNX7 and GFP–SNX30 decorated early endocytic structures that partially overlapped with EEA1 and SNX1, but did not correlate with APPL1 or CD63 (very early endosomal and late endosomal/lysosomal markers, respectively), suggesting that these SNX-BARs associate with intermediate stage endocytic structures (Fig. 3C,D), consistent with the previously reported distribution of SNX4 (Traer et al., 2007).

### Autophagic flux defects in SNX4-, SNX7- and SNX30-suppressed cells

**Fig. 4.**
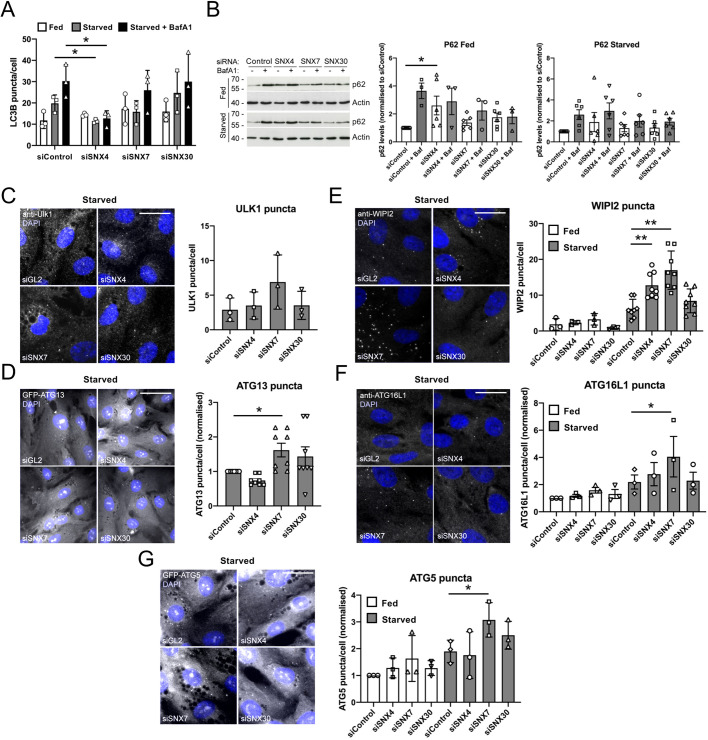
**The SNX4–SNX7 heterodimer is required for efficient autophagy.** (A) Autophagic flux analysis in hTERT-RPE1 cells silenced for SNX4, SNX7 or SNX30, and labelled for endogenous LC3B. Mean±s.d. of three experiments. (B) p62 turnover assay in hTERT-RPE1 cells silenced for SNX4, SNX7 or SNX30. Example blots to the left; quantitation to the right. Densitometry values were normalised against the siControl/BafA1 data, and are shown as mean±s.d. of ≥3 experiments. (C–G) Puncta analysis in SNX4, SNX7 and SNX30 siRNA treated hTERT-RPE1 cells labelled with antibodies or stably expressing GFP fusions of autophagy markers, with example images and quantitation shown as follows: (C) endogenous ULK1 puncta in starved cells; (D) GFP-ATG13 puncta in starved GFP–ATG13 stable cells; (E) endogenous WIPI2 puncta in fed and starved cells; (F) endogenous ATG16L1 puncta in fed and starved cells; (G) GFP–ATG5 puncta in fed and starved GFP–ATG5 stable cells. Mean±s.d. of ≥3 independent experiments, each counting >100 cells; example images show starved cells. *P<0.05; **P<0.01. Scale bars: 20 µm.

To test for the existence of an autophagy-specific SNX4-containing SNX-BAR heterodimer, we measured whether siRNA suppression of either *SNX7* or *SNX30* caused defects at the level of LC3B puncta formation. To analyse cells expressing only endogenous LC3B – and to clarify its impact on autophagic flux – we treated hTERT-RPE1 cells with the appropriate siRNA oligonucleotides before starving them in the absence or presence of BafA1, before fixing and staining with anti-LC3B antibodies (Fig. 4A). A significant reduction in LC3B puncta numbers was recorded in *SNX4*-silenced hTERT-RPE1s starved in the absence and presence of BafA1, suggesting that the reduced autophagy response was most likely due to the failure to assemble LC3B-positive autophagosomes, rather than an increase in the rate of flux (i.e. turnover of LC3 puncta) (Fig. 4A). Basal autophagy levels were similar between conditions (Fig. 4A). In these assays, the starvation-induced autophagy response in *SNX7*-silenced hTERT-RPE1 cells was consistently lower than in control and in *SNX30*-silenced cells, although this was not statistically significant (Fig. 4A). Finally, silencing of *SNX30* had no impact on the numbers of endogenous LC3B puncta in hTERT-RPE1s (Fig. 4A). Interestingly, suppression of SNX30 caused a significant increase in GFP–LC3B puncta in HEK293 cells (Fig. S1B). This might suggest that the SNX4–SNX30 heterodimer restricts autophagosome assembly in these cells, or that SNX30 suppression indirectly enhances LC3B lipidation by biasing assembly of an autophagy-enhancing SNX4–SNX7 heterodimeric complex. Combinatorial siRNA suppression of all three SNXs in GFP–LC3B HEK293 cells effectively phenocopied siRNA silencing of *SNX4*, with a significant decrease in LC3 puncta recorded (Fig. S1B).

To assess the consequences of suppression of SNX4, SNX7 and SNX30 on autophagic cargo flux, we carried out p62 (also known as SQSTM1) turnover assays in hTERT-RPE1 cells starved in the absence or presence of BafA1. In *SNX4*-silenced cells under basal conditions, p62 levels were significantly higher than in controls, and incubation in the presence of BafA1 did not increase p62 levels, indicative of a block in autophagic flux in full nutrients (Fig. 4B). Interestingly, in hTERT-RPE1 cells suppressed for either SNX7 or SNX30, the anticipated increase in p62/SQSTM1 levels following BafA1 treatment was also absent, although under these conditions, basal p62/SQSTM1 levels were not significantly different from controls (Fig. 4B). It was also notable that in *SNX4*-silenced hTERT-RPE1 cells, p62 levels did increase following addition of BafA1 during starvation, although this was not statistically significant (Fig. 4B). This suggests that when SNX4 levels are reduced, autophagic p62 turnover can still occur, but with reduced efficiency. Taken together, the interactions and functional data show that SNX4 can exist either as a weak homodimer, or as the core component of two distinct heterodimeric SNX-BAR complexes; SNX4–SNX7 and SNX4–SNX30. Which, if any, of these complexes acted selectively to regulate the autophagy process remained unclear.

### SNX4–SNX7 is an autophagy SNX-BAR heterodimer required during early stages of autophagosome assembly

Dynamic imaging studies have provided a model of quasi-hierarchical recruitment of autophagy regulators to the autophagosome assembly site, with both forward and reverse reinforcement interactions between key players (Karanasios and Ktistakis, 2015). To determine how the SNX4 homodimer and/or SNX4–SNX7/SNX4–SNX30 heterodimers influence autophagosome formation, the recruitment and retention of autophagy regulators at the autophagosome assembly site were assessed in hTERT-RPE1 cells by fluorescence microscopy (Fig. 4C–G). In this analysis, both reduced and increased autophagosome assembly site numbers can indicate a kinetic block in autophagosome assembly, when assessed alongside other tests for autophagy (e.g. LC3 puncta numbers or p62 turnover). For example, reduced numbers of assembly sites can be consistent with either a block in early signalling and/or the failure to recruit or stabilise early mediators at the autophagosome assembly site; by contrast, elevated numbers of assembly site foci can indicate enhanced autophagy signalling or assembly site stalling [as also seen in cells depleted for SNX18 (Knævelsrud et al., 2013)].

We began by analysing markers of the ULK1 kinase complex using anti-ULK1 antibodies and a stable GFP–ATG13 hTERT-RPE1 cell line following amino acid and growth factor withdrawal (Fig. 4C,D). Under these conditions, starvation-induced ULK1 and GFP–ATG13 puncta numbers were elevated only in SNX7-suppressed cells (although this was only statistically significant for GFP–ATG13; Fig. 4C,D). These data established for the first time a possible role for SNX7 in the regulation of autophagosome assembly dynamics, supporting the earlier hint that its suppression might be restricting LC3B lipidation in the same cell type (Fig. 4A). We next analysed subsequent stages of assembly site maturation – namely, PtdIns(3)P enrichment, and the recruitment and retention of the ATG8 lipidation machinery – using antibodies against WIPI2 and ATG16L1, and using the GFP–ATG5 stable hTERT-RPE1 cell line (MacVicar et al., 2015) (Fig. 4E–G). In common with the GFP–ATG13 data (Fig. 4D), we recorded significantly higher starvation-induced WIPI2, ATG16L1 and GFP–ATG5 puncta numbers in SNX7-silenced cells, confirming that autophagosome assembly is indeed sensitive to SNX7 levels in human cells (Fig. 4E–G). Starvation-induced WIPI2 puncta numbers were also significantly higher in cells suppressed for SNX4 (Fig. 4E,F); however, ATG16L1 and GFP-ATG5 puncta numbers did not differ significantly from controls in SNX4-suppressed cells (Fig. 4G). In starved hTERT-RPE1 cells, co-suppression of SNX4 with SNX7 elevated WIPI2 numbers above control levels, but co-suppression of SNX4 with SNX30 and SNX7 with SNX30 did not cause the same response (Fig. S1C), suggesting that changes in relative levels of cognate SNX4 dimers alters the autophagy response in complex ways.

**Fig. 5.**
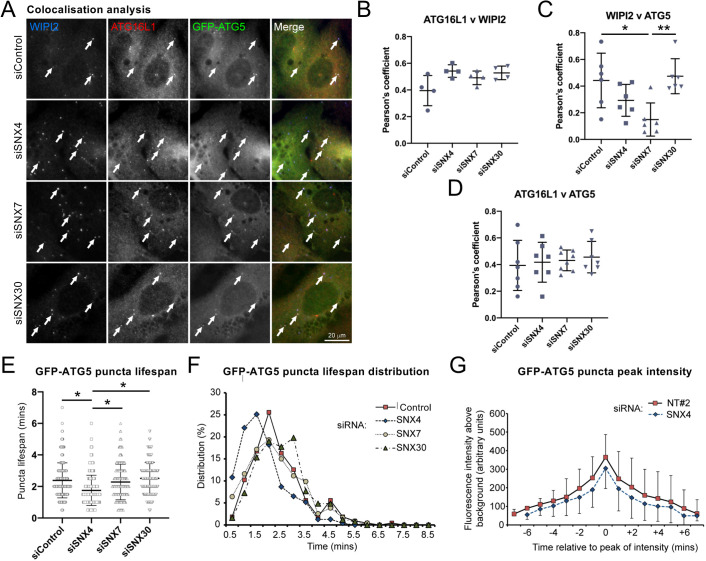
**SNX4/SNX7 depletion impacts on autophagy** **at the ATG5 recruitment/retention stage.** (A–D) Colocalisation analysis of early autophagosome markers in GFP–ATG5 stable hTERT-RPE1 cells. (A) Example images; arrows show examples of colocalising puncta. Scale bar: 20 µm. Pearson's coefficient analysis of colocalisation between: (B) ATG16L1 and WIPI2, (C) WIPI2 and ATG5 and (D) ATG16L1 and ATG5. Mean±s.d. of 4–8 cells over two separate experiments. (E) GFP–ATG5 puncta lifetime in stable hTERT-RPE1 cells silenced for SNX4, SNX7 or SNX30 during amino acid and growth factor starvation. Mean±s.d. of 130 (siControl), 124 (siSNX4), 144 (siSNX7) and 183 (siSNX30) puncta. (F) GFP–ATG5 puncta lifespan distribution in stable hTERT-RPE1 cells silenced for SNX4, SNX7 or SNX30 (showing percentage distribution). (G) Peak GFP–ATG5 puncta fluorescence intensity analysis in siControl and SNX4 siRNA-treated GFP–ATG5 stable hTERT-RPE1 cells. Mean±s.d. of ≥100 puncta analysed from two experiments. *P<0.05; **P<0.01.

Puncta analysis suggested that there are likely to be kinetic differences in the recruitment and/or retention of key autophagy markers at the isolation membrane during early autophagosome formation in SNX4- and SNX7-suppressed cells. To further assess where defects arose, we carried out colocalisation analysis of fixed GFP–ATG5 hTERT-RPE1 cells silenced individually for SNX4, SNX7 and SNX30, and labelled with anti-WIPI2 and anti-ATG16L1 antibodies (Fig. 5A). Each of these markers is recruited to the isolation membrane at a similar stage in advance of ATG8 lipidation. Analysis of ATG16L1 and WIPI2 hinted at increased colocalisation under all SNX4/7/30 siRNA conditions, although this was not statistically significant for any (Fig. 5C). Notably, however, colocalisation between WIPI2 and ATG5 was clearly lower in SNX4- and SNX7-suppressed cells (statistically significant only for SNX7; Fig. 5C). This suggests that effective ATG5 recruitment and/or retention at WIPI2-positive PtdIns(3)P-enriched early autophagic structures depends upon the presence of the SNX4–SNX7 heterodimer. Finally, we found no differences in ATG16L1 and ATG5 colocalisation when cells were suppressed for SNX4, SNX7 or SNX30, likely because of the high cytosolic background using these markers (Fig. 5A,D).

The absence of any specific defect at the level of ATG5 puncta numbers following SNX4 suppression (Fig. 4G) was surprising given the strong suppression of LC3B puncta numbers and elevated WIPI2 numbers recorded under this condition. The consequences of *SNX4* silencing on ATG5 during autophagosome assembly emerged during live-cell imaging experiments to assess the kinetics of assembly site assembly/disassembly in the GFP­–ATG5 hTERT-RPE1 cell line (Fig. 5E–G). In control siRNA cells, and in cells depleted for either SNX7 or SNX30, average GFP–ATG5 puncta lifespan was on average ∼2.5 min (Fig. 5E,F). By contrast, in *SNX4*-silenced cells, average GFP–ATG5 puncta lifespan was significantly shorter at ∼1.75 min (Fig. 5E), with the distribution of GFP–ATG5 puncta lifespans clearly altered (Fig. 5F). Furthermore, time-resolved comparisons revealed that in *SNX4*-silenced cells, GFP–ATG5 fluorescence intensities were consistently lower than in control cells – although this was not statistically significant at any individual time point (Fig. 5G). Together these data suggest that ATG5 recruitment and/or turnover kinetics are altered in SNX4-suppressed cells (Fig. 5D–G), and that cells most likely compensate for this by upregulating autophagosome assembly sites, meaning that steady state GFP–ATG5 puncta numbers appear similar to control cells (Fig. 4G).

### ATG9A trafficking is defective in SNX4 CRISPR knockout HeLa cells

**Fig. 6.**
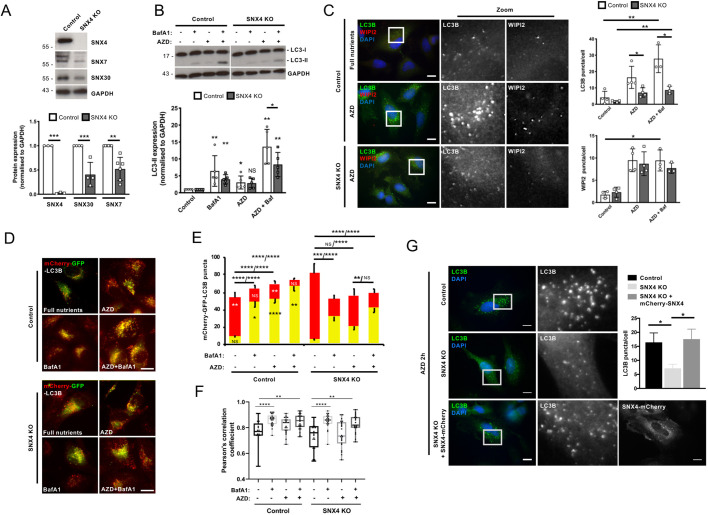
**Autophagy deficiency in a SNX4 CRISPR KO HeLa cell line.** (A) Immunoblotting analysis of SNX4, SNX7 and SNX30 levels in parental cells and SNX4 CRISPR KO clone A (for analysis of further clones, see Fig. S6A). Example blots above; quantitation below. Mean±s.d. (B) Immunoblotting-based LC3B lipidation analysis in the SNX4 CRISPR KO line treated with AZD8055 in the absence or presence of BafA1. Example blots above; quantitation below. Mean±s.d. (C) LC3B and WIPI2 puncta analysis in the SNX4 CRISPR KO line treated with AZD8055 in the absence or presence of BafA1 (for analysis of an additional clone, see Fig. S6B). Example images to the left; quantitation to the right. Boxes indicate regions shown in zoom images. Mean±s.d. of three experiments. (D) Example live-cell images of control and SNX4 KO HeLa cells stably expressing mCherry–GFP–LC3B, treated with BafA1, AZD8055, or BafA1 and AZD8055. (E) mCherry–GFP–LC3B flux assessment for the experiment described in D. Mean±s.d. of ∼20 cells per condition, imaged live. Asterisks superimposed upon the control bars represent statistical comparisons between control and SNX4 KO cells (one-way ANOVA); asterisks above the bars represent indicated pairwise comparisons for green/red analysis (Welch's *t*-test). (F) Colocalisation analysis (Pearson's correlation coefficient) of mCherry- and GFP-positive LC3B puncta in live control and SNX4 KO HeLa cells stably expressing mCherry–GFP–LC3B. Boxes indicate the interquartile range (IQR) and the horizontal bar marks the median. Whiskers indicate minimum and maximum values (Kruskal–Wallis non-parametric ANOVA). (G) Rescue of the autophagy (LC3B puncta) defect in SNX4 CRISPR KO HeLa cells transiently expressing mCherry–SNX4 and treated with AZD. Quantification shows mean±s.e.m. of three experiments. NS, not significant; *P<0.05; **P<0.01; ***P<0.001; ****P<0.0001. Scale bars: 10 µm.

Our siRNA-based results highlighted how the SNX4–SNX7 heterodimer acts during autophagosome formation at the ATG5 recruitment stage. During autophagosome assembly, the ATG12∼ATG5 conjugate is recruited via binding to ATG16L1, with additional membrane binding capability conferred by ATG5 itself (Romanov et al., 2012). To determine how the SNX4–SNX7 heterodimer influences this step, we used CRISPR-Cas9 to generate HeLa cell lines edited to eliminate SNX4 expression. Several clones were produced that showed reduced or absent SNX4 levels, and these were assessed for relative SNX7 and SNX30 expression (Fig. S6A,B). We selected clone ‘A’ for detailed analysis, because these cells showed depleted SNX7 and SNX30 alongside an absence of SNX4 (Fig. 6A; Fig. S6A,B). Analysis of the autophagy response in these cells following application of AZD for 2 h (Chresta et al., 2010) revealed a significant suppression of LC3B lipidation, and corresponding reduced numbers of LC3B-positive autophagosomes in SNX4 knockout (KO) cells in the absence and presence of BafA1 (Fig. 6B,C; see Fig. S6C for parallel data analysing SNX4 KO clone ‘B’). Interestingly, in contrast to *SNX4* siRNA-treated hTERT-RPE1 cells, in which WIPI2 puncta levels were significantly higher than in wild-type cells during autophagy stimulation (Fig. 4F,G), WIPI2 puncta numbers in SNX4 KO cells did not differ from wild type, with or without autophagy stimulation (Fig. 6C; Fig. S6C). This variability might be due to the different cell types tested (hTERT-RPE1 cells versus HeLa cells) and/or because of compensatory pathways emerging in the SNX4 KO cells. Interestingly, numbers of WIPI2 puncta were found to be lower in SNX18 KO cells than in wild-type cells following autophagy stimulation (Søreng et al., 2018), suggesting that these SNX-BARs influence different stages of autophagosome assembly. To assess autophagic flux, we generated mCherry–GFP–LC3B stable wild-type and SNX4 KO cells, and assessed autophagosome (red/green) and autolysosome (red only, because GFP is quenched at acidic pH) puncta numbers following 2 h AZD treatment in the absence or presence of BafA1 (Fig. 6D,E). Green/red-positive (yellow) autophagosome numbers were significantly fewer in the SNX4 KO cells than in controls under all treatment conditions except the basal state (i.e. treated with BafA1, AZD, or AZD and BafA1) (Fig. 6D,E). This suggests that autophagic flux is intact in SNX4 KO cells, but the efficiency of both assembly and flux is impaired. In fed SNX4 KO cells, LC3B-positive autolysosomes (red) were significantly more abundant than in control cells (Fig. 6E). This unexpected finding suggests that basal autophagic flux might be less efficient in the absence of SNX4, although this effect did not extend to cells following autophagy stimulation (Fig. 6E; the net reduction in LC3-positive puncta in SNX4 KO cells following autophagy stimulation possibly being caused by increased lysosomal clustering and/or fusion). As further evidence that loss of SNX4 affected autophagosome assembly, rather than lysosomal fusion (flux), we analysed colocalisation between red and green puncta under the same conditions (Fig. 6F). As expected, colocalisation increased when lysosomes were inhibited using BafA1 in control and AZD-treated cells (as a result of impaired GFP quenching), but no differences were observed in any single condition between control and SNX4 KO cells (Fig. 6F). Importantly, in SNX4 KO cells, transient expression of SNX4–mCherry was sufficient to rescue the autophagy defect at the level of LC3B puncta formation (Fig. 6G), arguing against off-target effects .

**Fig. 7.**
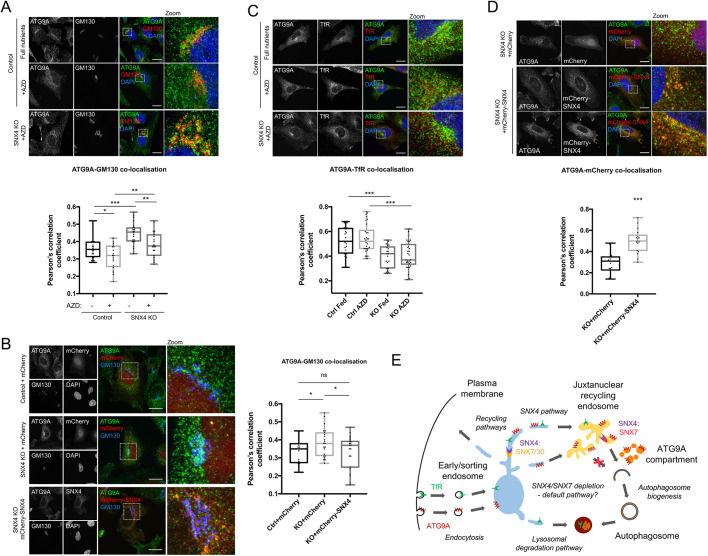
**Altered ATG9A trafficking in SNX4 CRISPR KO HeLa cells.** (A) ATG9A and GM130 colocalisation. Example images above; Pearson's correlation coefficient below, in control and SNX4 KO cells treated or not with AZD8055. (B) Rescue of the ATG9A Golgi localisation defect in AZD8055-treated SNX4 CRISPR KO cells using mCherry–SNX4. Example images to the left; Pearson's correlation to the right. (C) ATG9A and TfR colocalisation. Example images above; Pearson's correlation below, in control and SNX4 KO cells treated or not with AZD8055. (D) mCherry–SNX4 and ATG9A colocalisation in AZD8055-treated SNX4 CRISPR KO cells. Example images above; Pearson's correlation below. (E) Schematic of the possible roles for SNX4 during autophagosome assembly. In wild-type cells, SNX4 heterodimers coordinate cargo sorting in the early endosome and autophagy-stimulation dependent ATG9A mobilisation from the recycling endosome to facilitate autophagosome assembly. When SNX4 is suppressed, ATG9A reaches the juxtanuclear region via unidentified (‘default’) pathways; however, ATG9A is inefficiently mobilised from this localisation during autophagy under these conditions. Data points show individual cells from *n*≥3 experiments. Boxes indicate the interquartile range (IQR) and the horizontal bar marks the median. Whiskers indicate minimum and maximum values. ns, not significant; *P<0.05; **P<0.01; ***P<0.001. Scale bars:10 µm.

The multi-pass transmembrane protein ATG9A traffics through the endocytic network to establish a perinuclear RAB11/TGN-associated compartment from where it can be rapidly mobilised upon autophagy stimulation to form peripheral membrane pools required for efficient autophagosome biogenesis (for a recent discussion, see Shatz and Elazar, 2019). Because SNX18 has been found to coordinate ATG9A redistribution during autophagy via the actions of dynamin-2 (Søreng et al., 2018), we tested whether SNX4 also influences ATG9A trafficking during autophagy stimulation. We carried out immunofluorescence analysis of ATG9A localisation in cells counterstained for the Golgi marker, GM130 (also known as GOLGA2) (Fig. 7A,B), or transferrin receptor (TfR) (Fig. 7C). In wild-type HeLa cells, ATG9A steady state localisation comprised a prominent membrane pool focussed in the Golgi region, with additional dispersed vesicular profiles (Fig. 7A). As reported by others (Søreng et al., 2018; Young et al., 2006), autophagy stimulation (AZD treatment for 2 h) caused further dispersal of the ATG9A Golgi pool (Fig. 7A,B), whereas TfR localisation remained largely unchanged (Fig. 7C). At steady state (full nutrients, without AZD), ATG9A showed a significantly stronger colocalisation with GM130 in SNX4 KO cells when compared to controls (Fig. 7A), and whilst in both cell types ATG9A became more vesicular and dispersed following 2 h AZD treatment, ATG9A remained more closely associated with the Golgi region in the SNX4 KO cells, suggesting defective mobilisation of the ATG9A Golgi pool following autophagy stimulation (Fig. 7A). Importantly, the ATG9A redistribution defect could be rescued by transient expression of SNX4–mCherry in SNX4 KO cells (Fig. 7B). Interestingly, we noted some important differences in ATG9A behaviour between SNX4 KO and SNX18 KO cells (Søreng et al., 2018) with respect to TfR trafficking. Although ATG9A colocalisation with TfR was enhanced by SNX18 depletion, with TfR becoming more juxtanuclear in the absence of SNX18 (Søreng et al., 2018), in SNX4 KO cells, ATG9A colocalisation with TfR was significantly lower than in control cells and changed little following AZD treatment (Fig. 7C). Finally, and despite the lack of evidence for an interaction between SNX4 and ATG9A at the biochemical level (this was also true for other autophagy proteins; Fig. S7), SNX4–mCherry transiently expressed in the SNX4 KO background showed a strong colocalisation with ATG9A (Fig. 7D), suggesting that a subfraction of each protein resides in the same endomembrane compartment. Taken together, these data suggest that SNX4 contributes to the steady state localisation of ATG9A, and that SNX4 is required for efficient ATG9A redistribution upon autophagy stimulation to enable efficient autophagy responses. Our data are consistent with the SNX4–SNX7 autophagy SNX-BAR heterodimer contributing to the control of ATG9A trafficking from its steady state perinuclear localisation to the autophagosome assembly site during autophagy stimulation (Fig. 7E).

## DISCUSSION

Here, we have used an array of experimental protocols to describe how SNX4 acts as the core component of two heterodimeric complexes – SNX4–SNX7 and SNX4–SNX30 – that are associated with overlapping early endosomal compartments. These findings extend the concept of specific BAR domain-mediated associations within this family of proteins, complementing the known homodimeric interactions observed within the SH3 domain-containing SNX-BARs SNX9, SNX18 and SNX33 (Dislich et al., 2011; Haberg et al., 2008; van Weering et al., 2012; Park et al., 2010), and the heterodimeric assemblies that comprise the SNX-BAR membrane deforming ESCPE-1 coat complex, SNX1, SNX2, SNX5, SNX6 and SNX32 (Rojas et al., 2007; Simonetti et al., 2019; Wassmer et al., 2007, 2009). The molecular interactions that govern BAR domain homo- versus hetero-dimerisation remain opaque. Molecular modelling of SNX33 homodimers (Dislich et al., 2011), based on the published X-ray crystallographic structure of the SNX9 homodimer (Pylypenko et al., 2007), is consistent with the presence of a large dimer interface that contains a number of salt bridges, hydrophobic interactions, and hydrogen-bonding networks that together support tight homodimer formation (Dislich et al., 2011; Pylypenko et al., 2007). The lack of conservation between SNX9 and SNX33 in many of the amino acids that generate these interaction networks would appear sufficient to favour homo- over hetero-dimerisation in this context (Dislich et al., 2011). Consistent with this, molecular modelling and an X-ray crystallographic structure of the SNX1 homodimer has established how SNX1–SNX5 heterodimer formation arises (van Weering et al., 2012). One would predict, therefore, that the organisation of a similar network of interactions would favour the formation of the described SNX4 complexes, whilst disfavouring SNX7 and SNX30 homo- and hetero-dimers. Unfortunately, with the lack of sequence homology between the BAR domain of SNX1 and SNX9 and the corresponding domains in SNX4, SNX7 and SNX30, atomic resolution structures of the various SNX4 complexes will be required to describe how this dimer interface is organised.

In mammalian cells, SNX4 has been implicated in the tubular-based sorting of internalised TfR from early endosomes to the endocytic recycling compartment (Traer et al., 2007), and in the endosome-to-Golgi transport of ricin (Skånland et al., 2009, 2007). Our data reveal that a portion of SNX4 colocalises with ATG9A in the Golgi region, most likely at the RAB11 recycling endosome, and coordinates its trafficking to establish productive autophagosome assembly sites in mammalian cells. In yeast, Snx4 associates with the PAS (Zhao et al., 2016), via binding to Atg17 (Nice et al., 2002; Uetz et al., 2000), and supports efficient selective (Lynch-Day and Klionsky, 2010) and non-selective (Ma et al., 2018) forms of autophagy. We observed partial and transient correlations between SNX4 and markers of mammalian autophagosome assembly sites (Fig. 1D). Despite this, we did not find evidence for SNX4 association with the mammalian equivalent of Atg17 (FIP200, also known as RB1CC1; or indeed any other core autophagy protein), either by pulldowns (Fig. S7) or by quantitative SILAC proteomics (data not shown), suggesting that its influence on mammalian autophagy might be indirect. To determine where the defect in non-selective autophagy occurs in cells deficient for SNX4, we first carried out a series of imaging-based experiments in fixed and live cells siRNA silenced for *SNX4*, *SNX7* and/or *SNX30*, leading to two important observations: (i) that SNX4–SNX7 is the mammalian autophagy SNX4 heterodimer; and (ii) that the autophagy deficiency in SNX4- and/or SNX7-suppressed cells arises at the point of ATG5 recruitment, upstream of ATG8 (LC3) lipidation. In yeast, Snx4 acts with two additional SNX-BARs, Snx41 and Snx42, to mediate retrieval of the SNARE Snc1 from post-Golgi endosomes back to the late Golgi (Hettema et al., 2003). Given that yeast Snx42 is the expected SNX30 equivalent (Popelka et al., 2017), and with evidence that a Snx4–Snx42 dimer is needed for lipid homeostasis to support efficient autophagosome-to-vacuolar fusion (Ma et al., 2018), it is possible that yeast and mammalian cells differ in their mechanistic requirements for either SNX30/Snx42 or SNX7/Snx41 acting in concert with SNX4/Snx4 during autophagy. Indeed, evidence suggests that the yeast Snx4–Snx42 complex might act at later stages of autophagy (i.e. at the vacuolar fusion step; Ma et al., 2018), and/or specifically during selective autophagy, meaning that further studies focused on the possible roles of SNX30 are merited.

The molecular basis for the influence of the SNX4–SNX7 heterodimer during early stages of mammalian autophagy remains to be fully elucidated. Our data point strongly to a requirement for SNX4–SNX7 at the ATG12∼ATG5 conjugate recruitment stage, because we observed that GFP–ATG5 puncta are relatively short lived and generally less bright in cells lacking SNX4 when compared with puncta in control cells (Fig. 5E–G); meanwhile in SNX7-depleted cells, autophagosome assembly site markers including ATG13, ATG5, WIPI2 and ATG16L1 were clearly and dramatically amplified, arguing for a stalling of the autophagosome assembly pathway (Fig. 4C–G). Despite this, LC3B lipidation and/or puncta formation were only partially dampened following SNX7 suppression, perhaps indicating that increasing the abundance of less efficient assembly sites balances the suppressed autophagy response at the level of LC3B lipidation. Given that autophagic flux in SNX7- and SNX30-suppressed cells (as measured by p62 turnover kinetics; Fig. 4B) appeared to be mildly affected, it remains possible that these SNX-BARs have partially redundant autophagy roles in partnership with SNX4, at both assembly and maturation stages. This would be consistent with our observation that ATG5 puncta number and kinetics differed when comparing SNX4 and SNX7 siRNA suppressed cells, which would not be expected should they be acting wholly in concert and as the sole SNX4-containing mammalian autophagy heterodimer (Fig. 4G, Fig. 5E–G). However, different siRNA silencing efficiencies and the likely impact of an altered balance between SNX4–SNX7 and SNX4–SNX30 heterodimers when siRNA suppressing either partner SNX-BAR cannot be excluded.

The Simonsen laboratory has shown that another SNX-BAR, SNX18, acts as a positive regulator of mammalian autophagy (Knævelsrud et al., 2013; Søreng et al., 2018). SNX18 is targeted to the recycling endosome, from where it directs ATG16L1 and LC3-positive membrane delivery to the autophagosome assembly site (Knævelsrud et al., 2013). This role requires the membrane tubulation capabilities of SNX18, and is promoted by an LIR motif identified in the SH3 region of SNX18 (Knævelsrud et al., 2013). In further work, SNX18 was found to regulate autophagy via mobilisation of ATG9A-positive vesicles from the recycling endosome, with a requirement for SNX18 binding to dynamin-2 (Søreng et al., 2018). There are clear similarities between our characterisation of SNX4–SNX7-mediated autophagy and the involvement of SNX18 in the same pathway, although neither SNX4 nor SNX18 were identified as autophagy regulators in the respective, alternate siRNA-based screens (this study; Knævelsrud et al., 2013), perhaps due to cell line differences and/or siRNA efficiencies. At the level of autophagosome assembly kinetics, important differences were observed between these autophagy-regulating SNX-BARs. For example, whilst siRNA depletion of either SNX-BAR reduced the autophagy response in both studies, LC3 lipidation is enhanced following SNX18 overexpression (Knævelsrud et al., 2013), but was suppressed when SNX4 was overexpressed (Fig. 1F). Surprisingly, there are no clear differences in LC3 lipidation levels in SNX18 KO cells, except when assessed in full media in the presence of BafA1, although long-lived protein turnover is significantly reduced (Søreng et al., 2018). This differs from the LC3 lipidation defects observed in acute SNX18 siRNA-depleted cells (Knævelsrud et al., 2013), suggesting functional compensation may have arisen in the CRISPR null lines. Tellingly, this is also distinct from the scenario we describe in SNX4 siRNA-depleted and CRISPR KO cells, where LC3B lipidation was robustly suppressed in both cases. In starved SNX18 KO cells, WIPI2 and ATG16L1 puncta numbers are significantly lower (Søreng et al., 2018), whereas we observed that SNX4 and/or SNX7 siRNA suppression dramatically elevated WIPI2 and ATG16L1 puncta numbers during starvation. Further important differences were revealed upon analysis of ATG9A dynamics within SNX18 and SNX4 KO cells: whereas SNX18 KO increases colocalisation between ATG9A and TfR (Søreng et al., 2018), this was significantly reduced in SNX4 KO cells (Fig. 7C). Indeed, in SNX4 KO cells, ATG9A was more strongly associated with the Golgi region under basal conditions and during autophagy induction (Fig. 7A). Colocalisation between ATG16L1 and WIPI2 was observed to be weakened by SNX18 KO (although not statistically significant; Søreng et al., 2018), and interestingly, a similar situation was recorded in SNX7 siRNA-depleted cells, but at the level of WIPI2 versus ATG5 colocalisation (Fig. 5A,C). Evidently, these autophagy-regulating SNX-BARs have common, yet distinct influences at the ATG16L1 and/or ATG5 recruitment stage; a finding that is consistent with the autophagy defects observed in ATG9A KO cells (Orsi et al., 2012).

In summary, our data implicate the SNX4–SNX7 heterodimer in efficient autophagosome biogenesis in mammalian cells. In the absence of either partner, autophagosome membrane expansion is impaired at the level of ATG5 recruitment and/or stability at autophagosome assembly sites. When SNX4 is suppressed, ATG9A associates more strongly in the Golgi region than observed in wild-type cells, suggesting altered membrane distribution or sorting. Here, ATG9A colocalises with SNX4, and we suggest that this is evidence of dual protein-sorting roles for SNX4–SNX7 in mammalian cells, namely: (i) selective cargo sorting away from the degradation route at the level of the peripheral early endosome (e.g. TfR; Traer et al., 2007); and (ii) ATG9A mobilisation from the juxtanuclear recycling compartment (Fig. 7E). This provides another example of endocytic involvement during autophagosome assembly, and highlights the dynamic nature of interrelationships between organelles during the autophagosome biogenesis pathway.

## MATERIALS AND METHODS

### Materials and antibodies

All materials were purchased from Sigma unless otherwise stated. BafA1 (B1793); AZD8055 (Selleckchem, S1555); puromycin (P8833); DAPI (D121490). The following antibodies were used: anti-GAPDH (G8796; 1:2000 IB); anti-ATG9A (Abcam, ab108338; 1:2000 IB; 1:200 IF); anti-ATG16L1 (MBL, PM040; 1:1000 IB; 1:100 IF); anti-LC3B (L7543; 1:1000 IB; 1:400 IF); anti-LC3B (MBL, PM036; 1:100 IF); anti-p62/SQSTM1 (Abnova, H00008878; 1:1000 IB); anti-WIPI2 (BioRad, MCA5780GA; 1:400 IF); anti-ULK1 (Cell Signaling, D8H5/8054; 1:1000 IB); anti-ATG7 (Cell Signaling, D12B11/8558; 1:1000 IB); anti-ATG3 (Cell Signaling, 3415; 1:1000 IB); anti-FIP200 (SAB4200135; 1:500 IB); anti-GFP (Covance, MMS-118P; 1:2000 IB); anti-GM130 (Santa Cruz, sc216; 1:200 IF); anti-SNX4 (Abcam, ab198504; 1:2000 IB); anti-SNX30 (Abcam, ab121600; 1:2000 IB); anti-SNX7 (Proteintech, 12269-1-AP; 1:1000 IB); anti-SNX1 (BD Biosciences, 611482; 1:1000 IB); anti-SNX2 (BD Biosciences, 611308; 1:1000 IB); anti-SNX5 (Proteintech, 17918-AP; 1:1000 IB); anti-SNX6 (Santa Cruz, sc-8679; 1:1000 IB); anti-SNX7 (Abcam, Ab37691; 1:1000 IB); anti-EEA1 (BD Biosciences, 610456; 1:1000 IB); anti-CD63 (Santa Cruz, sc-51662; 1:1000 IB); anti-APPL1 (kind gifts from Pietro De Camilli, Yale University and Philip Woodman, University of Manchester; 1:100 IF); anti-β-actin (A1978; 1:1000 IB); anti-TfR (Santa Cruz, sc-65882; 1:2000 IB; 1:200 IF); anti-mouse HRP (Stratech, G32-62DC-SGC; 1:10,000 IB); anti-rabbit HRP (Stratech, G33-62G-SGC; 1:10,000 IB); Alexa Fluor 488 (Invitrogen, A-11029/A-11034; 1:300 IF); Alexa Fluor 568 (Invitrogen, A-11031/A11036; 1:300 IF); Alexa Fluor 647 (Invitrogen, A-21236/A-21244; 1:300 IF).

### Cell lines and cell culture

Parental HeLa and hTERT-RPE1 cells, GFP–ATG13 RPE1 (this study), GFP–ATG5 RPE1 (MacVicar et al., 2015), YFP–LC3B RPE1 (this study), GFP–SNX4 HeLa (this study), GFP–SNX4 hTERT-RPE1 cells (this study), GFP–LC3B HEK293 (a gift from Sharon Tooze, Francis Crick Institute, London, UK) stable cell lines, and the SNX4 CRISPR null HeLa cells (including wild-type and CRISPR KO cells stably expressing mCherry–GFP-LC3B; this study) were maintained in DMEM containing 4500 mg/ml glucose (Sigma, D5796), supplemented with 10% FBS (Sigma, F7524) [and with 1% penicillin-streptomycin (Sigma, P4333) for CRISPR null cell line cloning and maintenance only]. Cells were grown at 37°C with 5% CO_2_, and were tested routinely for contamination (e.g. mycoplasma). Cells were starved for 1 h in starvation medium (140 mM NaCl, 1 mM CaCl_2_, 1 mM MgCl_2_, 5 mM glucose, and 20 mM Hepes, pH 7.4) (Axe et al., 2008).

### Vector design and cloning of SNX-BARs, viruses and transductions

The SNX-BAR genes were amplified from a HeLa cell cDNA library using conventional PCR. For transient transfection of mammalian cells, the SNX-BAR genes were cloned into pEGFP.C1 and pmCherry.C1 vectors (Clontech), for the transduction of mammalian cells the genes were cloned into EGFP/mCherry.C1 pXLG3 lentivector system (a gift from Giles Cory, University of Bristol, UK). Lentiviruses were generated in HEK293T cells by transfection with cDNAs along with packaging vectors pMD2G and pAX2 (a gift from Giles Cory, University of Bristol, UK). Lentiviral particles were collected at 48 h, cleared by centrifugation (2900 ***g***, 10 min), then passed through a 0.45 µm polyethersulfone filter to be used immediately or stored at −80°C. Control or SNX4-suppressed HeLa cells overexpressing mCherry or mCherry–SNX4 were generated as follows: cells were plated in 6-well plates and transduced with the corresponding lentiviruses. After 3 d, cells were seeded on coverslips for the corresponding experiment. CFP–LC3B was generated from GFP–LC3B (Lane lab) by colour switching between pEGFP and pECFP vectors.

### CRISPR/Cas9 for generation of SNX4 KO cells

The CRISPR-Cas9 plasmid (pX330) developed by the Zhang laboratory (Broad Institute, Cambridge, MA) was used for targeted gene knockout (Cong et al., 2013). The sequences for the gRNAs (5′-GCGGTCGGCAAGGAAGCGGA-3′) were calculated using the online tool from the Zhang laboratory and cloned in pX330 accordingly (www.genome-engineering.org). To generate SNX4 knockout cells, HeLa cells were co-transfected with CRISPR-Cas9 plasmids and a plasmid conferring puromycin resistance using FuGENE (Promega, E2693). 24 h after transfection, cells were subjected to 24 h of puromycin selection (Sigma, P8833; 2 µg/ml), after which cells were resuspended using Accutase (BioLegend, 423201) and diluted in Iscove's Modified Dulbecco's Medium (Sigma, I3390) supplemented with 10% FBS to 3.5 cells/ml. Subsequently, 200 μl cell suspension was plated in each well of ten 96-well plates, and after 3 weeks the plates were screened for the presence of cell colonies. Colonies were expanded in DMEM and subjected to lysis and western blotting to determine the expression levels of the target protein.

### Immunoblotting

Cells grown on 6-well plates were initially washed with ice-cold PBS, then lysed with 200 µl/well ice-cold radioimmunoprecipitation assay (RIPA) buffer consisting of 50 mM Tris-HCl (pH 7.4), 1% Triton X-100 (Sigma, 9002-93-1), 0.5% sodium deoxycholate (Sigma, D6750), 150 mM NaCl (Sigma, S9888), 0.1% SDS (Sigma, 436143) and supplemented with one tablet of protease inhibitor (Roche, 4693159001) per 10 ml of RIPA buffer. The homogenates were incubated on ice for 10 min, then cleared by centrifugation at 12,000 ***g*** for 15 min at 4°C. Supernatants were collected as soluble fractions. Proteins were transferred to nitrocellulose membranes (Bio-Rad, 1620115), and membranes were then incubated overnight with primary antibody diluted in 5% milk or 2.5% BSA in TBS buffer containing Triton X-100. Primary and secondary antibodies used are listed above. Membranes were then washed three times prior to incubation with ECL chemiluminescence reagents (Geneflow, K1-0170), and band intensities were detected on film (GE Healthcare, 28906837).

### GFP-Trap

For GFP-Trap immunoisolation, cells in 10-cm plates were washed with ice cold PBS, and 500 µl of lysis buffer (50 mM Tris-HCl, 0.5% NP40, 1 mM PMSF, 200 µM Na_3_VO_4_, protease inhibitors, pH 7.5) was added. Cells were scraped and lysates collected and incubated on ice for 10 min. Lysates were cleared by centrifugation at 20,000 ***g*** for 10 min at 4°C and added to pre-equilibrated beads (Chromotek, GTA-100) then rotated for 2 h at 4°C. The sample was then spun at 2700 ***g*** for 2 min at 4°C to pellet beads, which were washed three times in wash buffer (50 mM Tris base; 1 mM PMSF; 200 µM Na_3_VO_4_; protease inhibitors) then resuspended in SDS–PAGE gel sample buffer.

### Immunoprecipitations

Confluent HEK293 cells in 15-cm dishes were washed with ice-cold PBS at least three times prior to lysis. Cells were lysed with 500 µl of ice-cold lysis buffer (50 mM Tris-HCl, 0.5% NP-40 and Roche protease inhibitor cocktail, pH7.5) and lysis was aided through the use of a cell scraper. Cell lysates were cleared by centrifugation in a bench-top centrifuge for 10 min at 13,000 rpm (12,000 ***g*)** at 4°C. Cleared lysates were incubated with 2 µg of either IgG control antibody or SNX1, SNX4, SNX7 and SNX30 antibody overnight at 4°C on a roller. 25 µl of cleared lysate was retained as 5% total protein input. Protein G sepharose beads (GE Healthcare) were washed three times in lysis buffer to remove any residual ethanol from the storage buffer. Pre-equilibrated Protein G sepharose was then added to Eppendorf tubes containing the lysate/antibody mixture and these tubes were further incubated for 1 h at 4°C on a roller. After incubation, the beads were pelleted by centrifugation at 4°C for 30 s at 4000 rpm (1073 ***g*)**. The beads were then washed three times in 1 ml of lysis buffer. Finally, all buffer was removed and Protein G beads (and associated immunoprecipitated proteins) were either stored at −20°C or processed immediately for SDS–PAGE and western blotting.

### siRNA suppression

The siControl oligonucleotide used for experiments in Fig. 1A and Fig. S1B was: 5′-GACAAGAACCAGAACGCCA-3′. For all other experiments, the siControl oligonucleotide was: 5′-GUACGCGGAAUACUUCGAUU-3′. The following siRNA oligonucleotides were used for experiments targeting SNX4, SNX7 and SNX30 proteins in autophagy (Dharmacon, siGENOME or SMARTpool): ATG5, 5′-GGAAUAUCCUGCAGAAGAA-3′; SNX4 SMARTpool D1, 5′-UUACUGACCUUAAGCACUA-3′; D2, 5′-GAAACAAGGUCAGUUGAAC-3′; D3, 5′-GCGGCGAUAUAGUGAAUUU-3′; D4, 5′-GCGACGGAUUGGUUUAGAA-3′; SNX7 SMARTpool D1, 5′-GCGGAUGUCUGGACUCUCA-3′; D2, 5′-GUACGUGCUUUAUAGUGAA-3′; D3, 5′-GGAGACGAUAUCAAGAUUU-3′; D4, 5′-GCACACCCCACUCUGAUUA-3′; SNX30 SMARTpool D1, 5′-ACAAGAACAUCCAGUAUUA-3′; D2, 5′-GAAGAAGAGGGACCAAGUU-3′; D3, 5′-CGGCGGACGUCGAGAAAUG-3′; D4, 5′-GGAGUCGAUUAUUCCACUA-3′; SNX4 3′UTR, 5′-GAUCCACUAAUCUGUUAUA-3′.

### Immunofluorescence and cell imaging

For fixed cell imaging, cells were seeded on coverslips, washed twice with PBS and incubated with 4% formaldehyde for 15 min or −20°C methanol for 5 min. Cells were then incubated for 30 min with primary antibodies (listed above) in PBS. Cells were washed three times with PBS and incubated with secondary antibodies (listed above) and counterstained with DAPI (100 ng/ml) for 10 min. Cells were then washed again with PBS and mounted in Mowiol containing 25 mg/ml DABCO (1,4-Diazabicyclo [2.2.2] octane; D27802). Fixed-cell images were obtained using an Olympus IX-71 inverted microscope (60× U Plan Fluorite objective; 0.65–1.25 NA, oil immersion lens) fitted with a CoolSNAP HQ CCD camera (Photometrics, AZ) driven by MetaMorph software (Molecular Devices). Confocal microscopy was carried out using a Leica SP5-AOBS confocal laser scanning microscope (63× oil immersion objective, 1.4 NA; or 100× oil immersion objective, 1.4 NA) attached to a Leica DM I6000 inverted epifluorescence microscope. Laser lines were: 100 mW Argon (for 458, 488 and 514 nm excitation), 2 mW Orange HeNe (594 nm) and 50 mW diode laser (405 nm). The microscope was run using Leica LAS AF software (Leica, Germany). MetaMorph software was used to quantify puncta numbers. A TopHat morphology filter was used to score circular objects of 5 pixels (∼1 µm) diameter. An automated cell count was then performed to count the number of selected items. For a typical experiment, 15 random fields were imaged and puncta numbers per cell in each field were counted. Colocalisation was determined by acquiring images from ∼25 cells per condition, with Pearson's coefficient calculated using Fiji software.

### Directed yeast two-hybrid screens

The yeast strain AH109 was co-transfected with bait vector full-length human SNX4 or Lamin cloned into pGBKT7 (Clontech, Oxford, UK) against a prey library of FL SNX-BARs. Yeast clones with positive bait–prey interactions were selected on SD –Leu –Trp plates supplemented with 1 mM 3-AT (3-amino-1,2,4-triazole) and α-X-Gal (Glycosynth, Cheshire, UK).

### Statistical analysis

Graphical results were analysed with GraphPad Prism 7 (GraphPad Software, San Diego, CA), using an unpaired Student's *t*-test (where not specified), or a Kruskal–Wallis test for non-parametric ANOVA. Results are expressed as mean±s.e.m. or mean±s.d., as indicated.

## Supplementary Material

Supplementary information

Reviewer comments
