# Supplementary material for: A heterodimeric SNX4­–SNX7 SNX-BAR autophagy complex coordinates ATG9A trafficking for efficient autophagosome assembly
Source: J Cell Sci. 2020 Jul 15;133(14):jcs246306. doi: 10.1242/jcs.246306 (PMC7375690; doi:10.1242/jcs.246306)
Supplement: Supplementary information [file joces-133-246306-s1.pdf]

SUPPLEMENTAL FIGURES

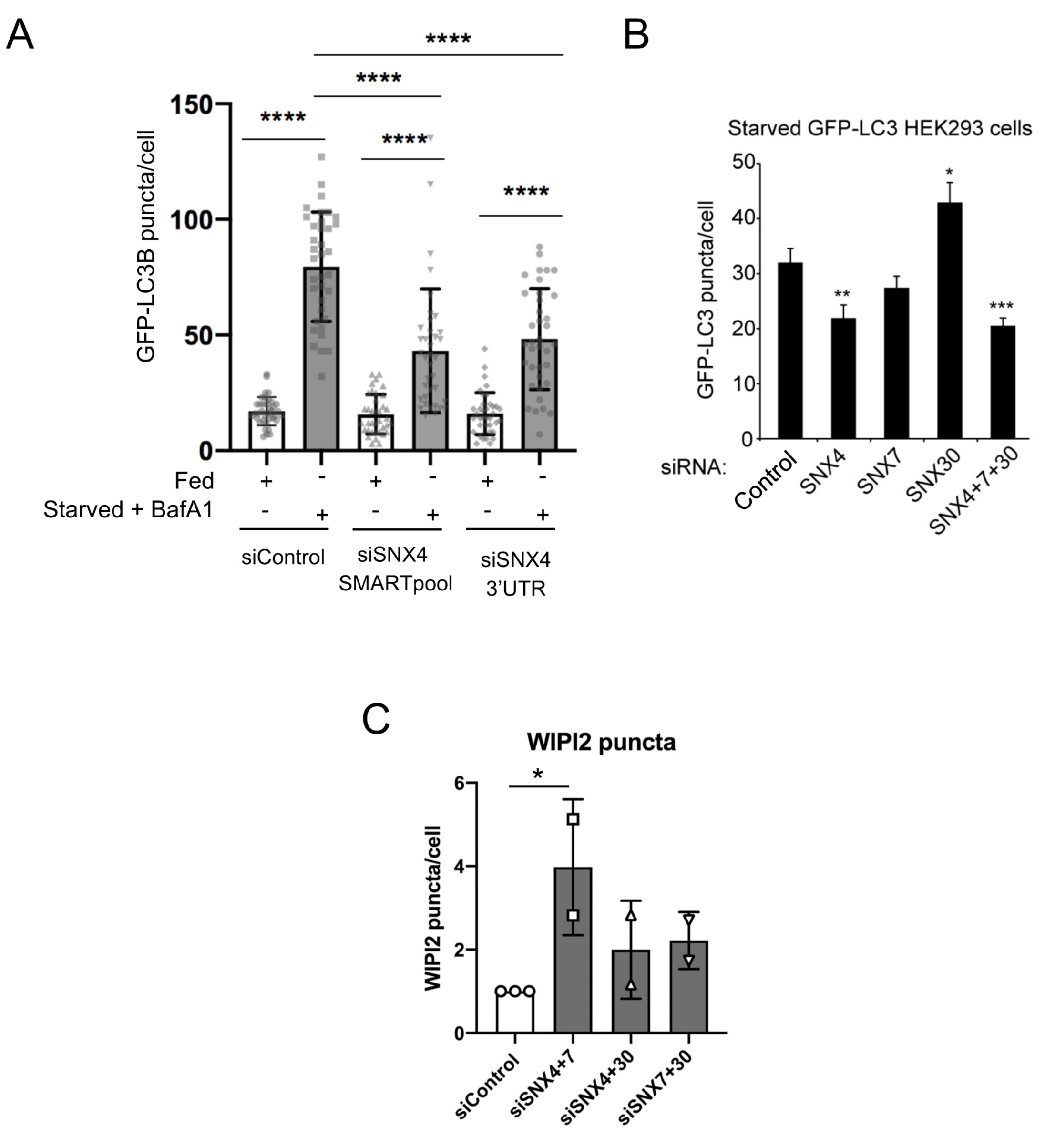

Figure S1: LC3B puncta analysis using various siRNA oligos. (A) GFPi LC3B puncta analysed after 1 h starvation in the presence of BafA1, in hTERTi RPE1 cells treated with siSNX4 SMARTpool or siSNX4 3'UTR siRNA. Data points represent GFPi LC3B puncta in individual cells. (B) GFPi LC3B puncta in HEK293 cells treated with siSNX4, siSNX7, siSNX30, or a combination of all 3, starved for 1 h. (C) WIPI2 puncta counts in starved hTERT RPE1 cells silenced as shown. *p<0.05B **p<0.01B ***p<0.001****p<0.0001.

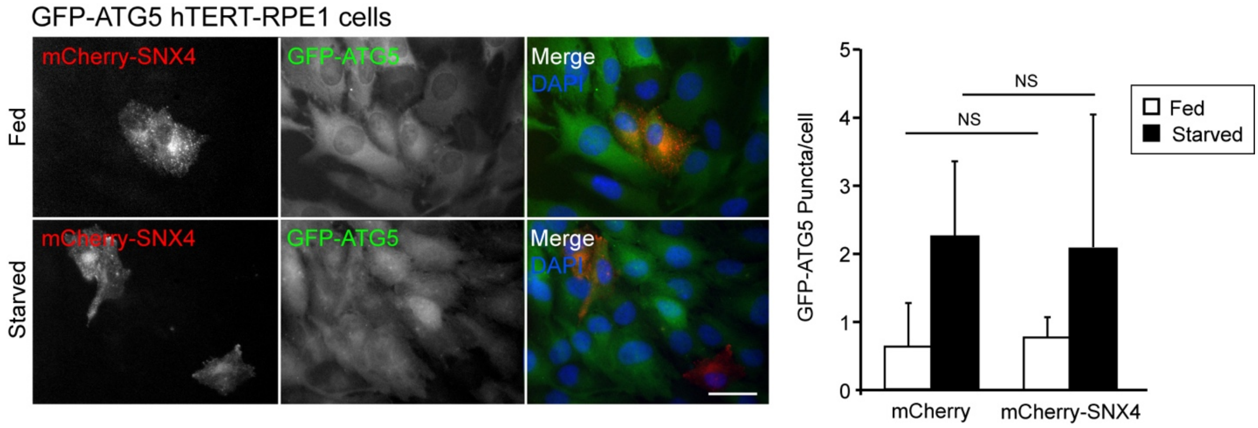

Figure S2: Steady state ATG5 puncta numbers are not altered in cells overexpressing SNX4. ATG5i GFP hTERT RPE1 cells were transiently transfected with mCherryi SNX4 (mCherry as control), and ATG5 puncta numbers were counted using automated software (MetaMorph) in fed and starvation conditions (1 h). Example images of mCherryi SNX4 fields to the leftB quantitation to the right. Mean ± SDB n=3B Bar = 20 μm.

Journal of Cell Science • Supplementary information

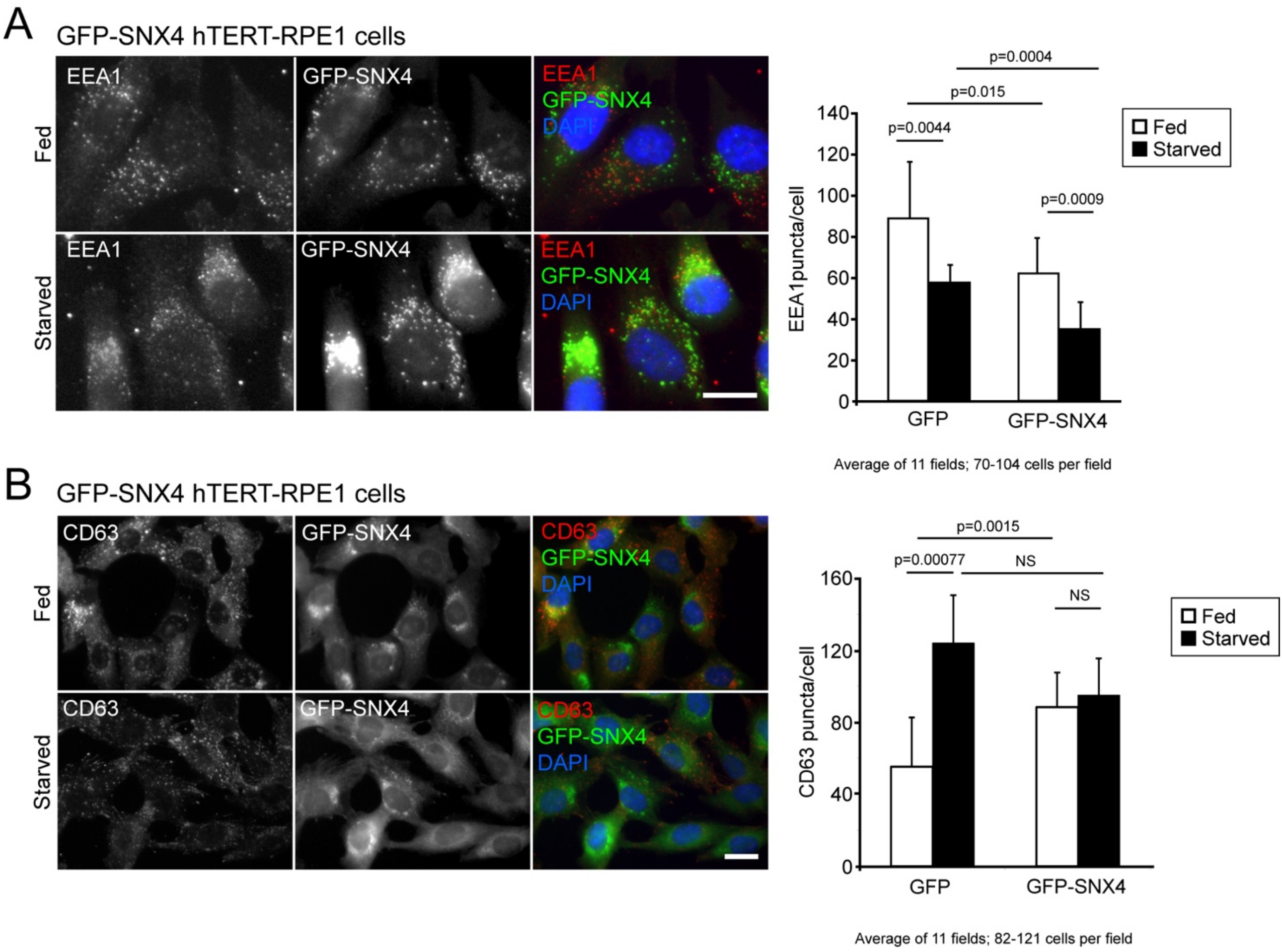

Figure S3: GFPi SNX4 stable hTERT RPE1 cells show defects in endolysosomal compartments. (A) hTERT RPE1 cells stably overexpressing GFPi SNX4 were starved (1 h), then fixed and stained with antibodies against the (**A**) early endosome (EEA1) and (**B**) the lysosome (CD63). Example images to the leftB quantitation to the right. Means ± SDB p values are shown on the graphs. Bars = 10 µm.

Journal of Cell Science • Supplementary information

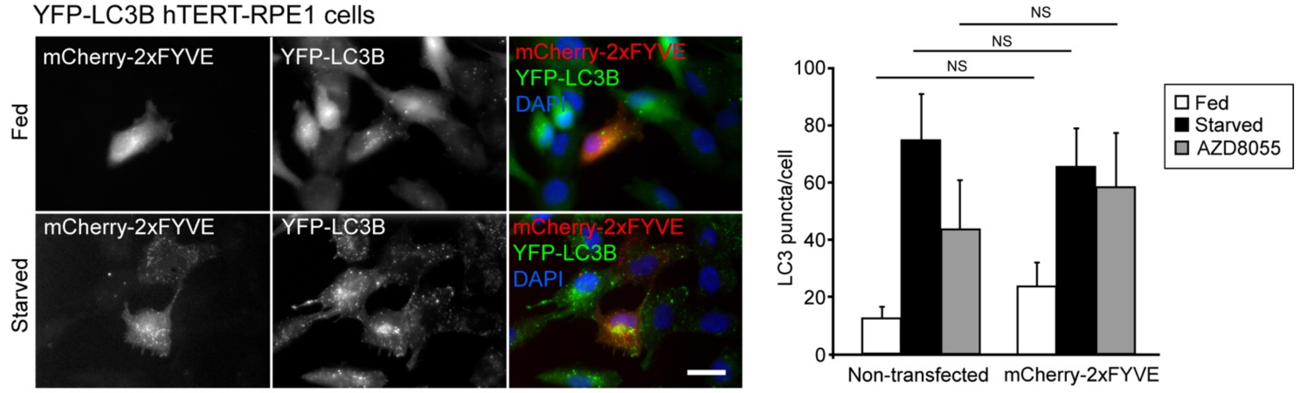

Figure S4: Overexpression of 2xFYVE does not influence the autophagy response. hTERT RPE1 cells stably expressing YFP-LC3B were transiently transfected with mCherry-2xFYVE, and LC3B punta were counted following starvation or AZD8055 treatment (1 h) in transfected and untransfected cells. Example images of fed and starved cells to the left; quantitation to the right. Bar = 10 µm.

Journal of Cell Science • Supplementary information

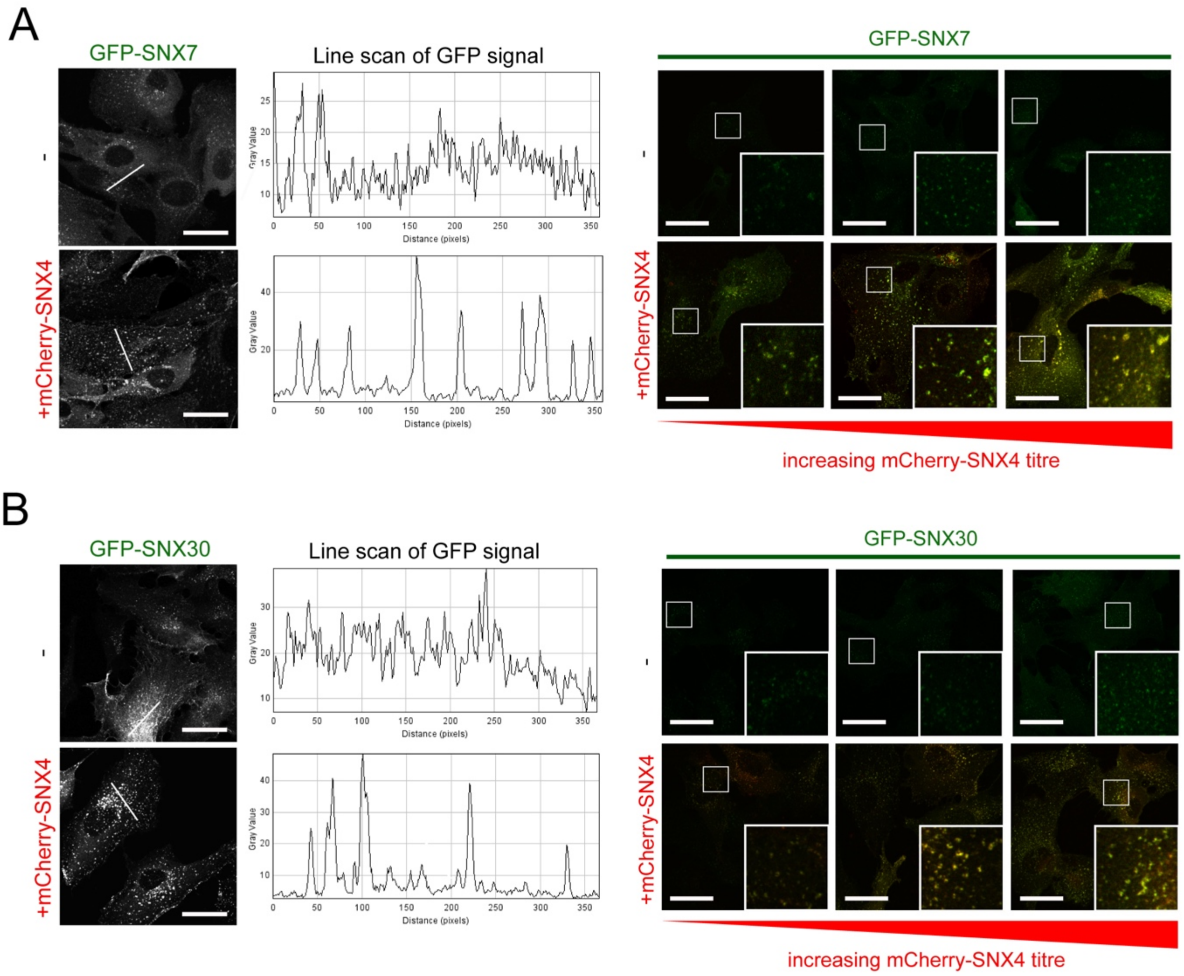

Figure S5: GFP-SNX7 and GFP-SNX30 display predominant cytoplasmic localisations when expressed alone; however, when co-expressed with mCherry-SNX4 they show a punctate localisation that correlates with SNX4 levels. RPE1 cells were lentivirally transduced with GFP-SNX7 (**A**) or GFP-SNX30 (**B**), alone or in combination with increasing titres of mCherry-SNX4. Transduced cells were then fixed and imaged. Line scans of GFP signal show that GFP-SNX7 and GFP-SNX30 localise on cytosolic puncta only when the heterodimeric partner SNX4 is co-expressed. Bar = 20 μm.

Journal of Cell Science • Supplementary information

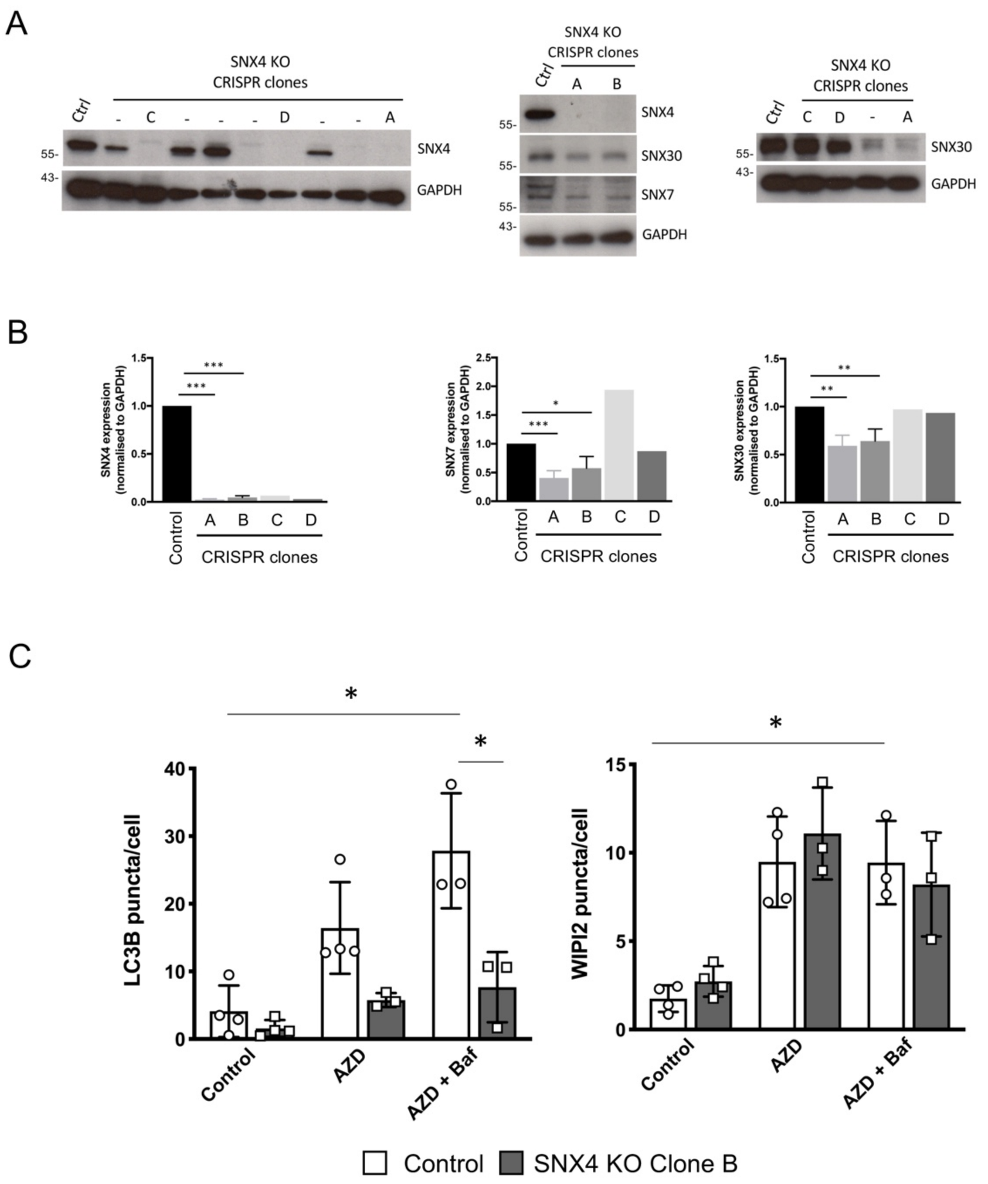

Figure S6: Analysis of SNX4 CRISPR clones. Individual colonies of HeLa cells following SNX4 CRISPRi Cas9 treatment were selected and expanded. (A) Example blots for SNX4, SNX7, and SNX30. (B) Collated quantitation of clones Ai D. (C) LC3 and WIPI2 puncta counts after AZD treatment (2 h, +/i BafA1) in clone "B" cells SNX4 CRISPR KO cells. NoteB control reference data are the same as used in Fig. 5C in the main text. *p<0.05B **p<0.01B ***p<0.001.

Journal of Cell Science • Supplementary information

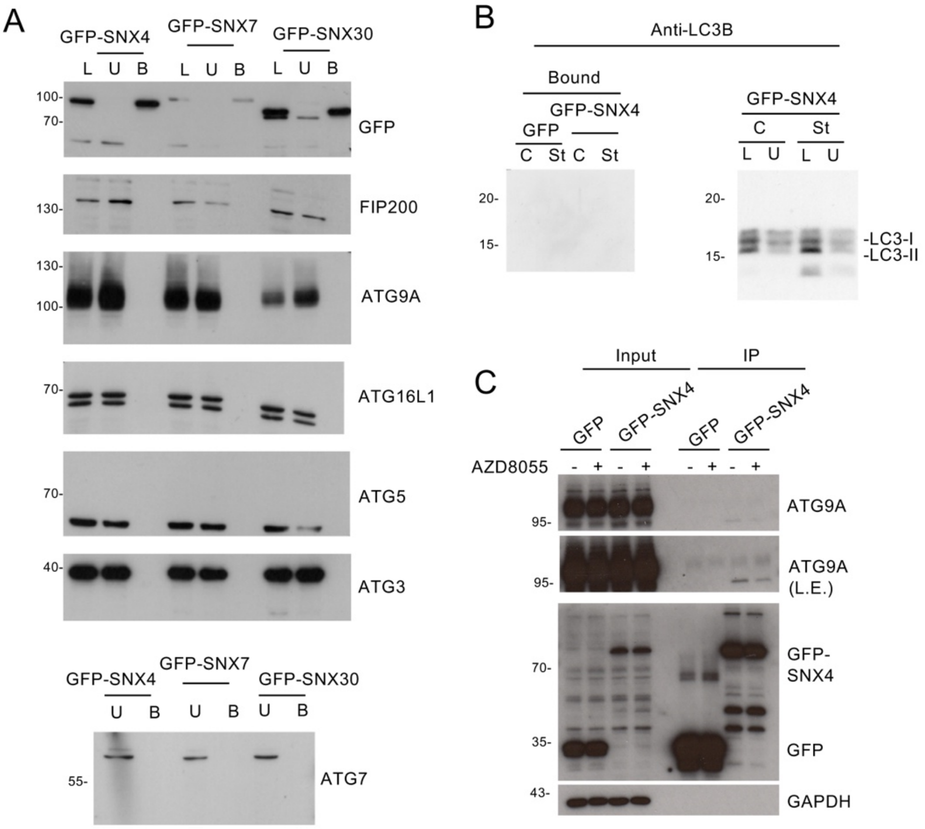

Figure S7: SNX4/7/30 do not coi precipitate with known autophagy proteins. (**A**) HeLa cells expressing GFPi SNX4/7/30 were lysed and subjected to GFPi TRAP immunoprecipitation. Lysates (L), unbound fractions (U) and bound fractions (B) were blotted for the autophagy markers shown. (**B**) GFPi SNX4 HeLa cells were starved (1 h), fractionated, and lysates used for GFPi TRAP immunoprecipitation. Samples were blotted for LC3B, which was not detected in the bound fractions for GFPi SNX4 or GFP (control) lysates. (**C**) GFPi SNX4 cells were treated with AZD8055, lysed, and subjected for GFPi TRAP immunoprecipitation. Inputs and bound fractions were blotted for ATG9A. (L.E. = long exposure).
