# Supplementary material for: A heterodimeric SNX4­–SNX7 SNX-BAR autophagy complex coordinates ATG9A trafficking for efficient autophagosome assembly
Source: J Cell Sci. 2020 Jul 15;133(14):jcs246306. doi: 10.1242/jcs.246306 (PMC7375690; doi:10.1242/jcs.246306)
Supplement: Reviewer comments [file joces-133-246306_review_history.pdf]

Journal of Cell Science

A heterodimeric SNX4–SNX7 SNX-BAR autophagy complex coordinates ATG9A trafficking for efficient autophagosome assembly

Zuriñe Antón, Virginie M. S. Betin, Boris Simonetti, Colin J. Traer, Naomi Attar, Peter J. Cullen and Jon D. Lane
DOI: 10.1242/jcs.246306

Editor: Tamotsu Yoshimori

Review timeline

Original submission:	15 March 2020
Editorial decision:	9 April 2020
First revision received:	29 May 2020
Accepted:	2 June 2020

Original submission

First decision letter

MS ID#: JOCES/2020/246306

MS TITLE: A heterodimeric SNX4:SNX7 SNX-BAR autophagy complex coordinates ATG9A trafficking for efficient autophagosome assembly

AUTHORS: Dr Zurine Anton, Virginie Betin, Boris Simonetti, Colin Traer, Naomi Attar, Peter Cullen, and Jon Lane
ARTICLE TYPE: Research Article

We have now reached a decision on the above manuscript.

To see the reviewers' reports and a copy of this decision letter, please go to: https://submit-jcs.biologists.org and click on the 'Manuscripts with Decisions' queue in the Author Area. (Corresponding author only has access to reviews.)

As you will see, the reviewers raise a number of substantial criticisms that prevent me from accepting the paper at this stage. They suggest, however, that a revised version might prove acceptable, if you can address their concerns. If you think that you can deal satisfactorily with the criticisms on revision, I would be pleased to see a revised manuscript. We would then return it to the reviewers.

We are aware that you may currently be unable to access the lab to undertake experimental revisions. If it would be helpful, we encourage you to contact us to discuss your revision in greater detail. Please send us a point-by-point response indicating where you are able to address concerns raised (either experimentally or by changes to the text) and where you will not be able to do so within the normal timeframe of a revision. We will then provide further guidance. Please also note that we are happy to extend revision timeframes as necessary.

Please ensure that you clearly highlight all changes made in the revised manuscript. Please avoid using 'Tracked changes' in Word files as these are lost in PDF conversion.

I should be grateful if you would also provide a point-by-point response detailing how you have dealt with the points raised by the reviewers in the 'Response to Reviewers' box. Please attend to

© 2020. Published by The Company of Biologists under the terms of the Creative Commons Attribution License (https://creativecommons.org/licenses/by/4.0/).

all of the reviewers' comments. If you do not agree with any of their criticisms or suggestions please explain clearly why this is so.

Reviewer 1

Advance summary and potential significance to field

The manuscript by Anton et al. entitled “A heterodimeric SNX4:SNX7 SNX-BAR autophagy complex coordinates ATG9A trafficking for efficient autophagosome assembly” reports on a role for heterodimer SNX4:SNX7 in the autophagosome formation. The authors confirm that SNX4 is a core component of two heterodimers (SNX4:SN7 and SNX4:SNX30) (as previously shown (Van Weering et al., 2012). They present further evidence that both complexes localize to early endosomes and that depletion of SNX4 prevents efficient lipidation of LC3B and leads to a loss of LC3 puncta both in fed and in starved conditions. To elucidate the role of the SNX4 complexes in autophagy, they do a series of siRNA experiments to analyze how depletion of the different components affects numbers and localization of early and late autophagy markers and conclude that the SNX4:SNX7 complex regulates ATG9 trafficking. This is a potentially interesting study as it represents a novel role for the heterodimer SNX4:SNX7 in autophagy. However, the data presented in the manuscript must be strengthened to support the conclusions made, as indicated below.

Comments for the author

General comments:
1. The authors use several different cell lines throughout the manuscript, which in principle is fine (and a strength) as long as the results obtained in one cell line is confirmed in another cell line. Here however, it seems like the authors have done different experiments in different cell lines, which could give the impression that one has cherry-picked results to make a story. They have used hTERT-immortalized retinal pigment epithelial (hTERT-RPE1) cells for most of the initial autophagy experiments, but then use HeLa cells to IP the SNX4 complexes and generate a CRISPR-Cas9 knock out of SNX4 and also HEK293 GFP-LC3 cells. They state “This probably reflects cell-type specific differences in the relative balance of SNX4-containing heterodimers and/or the efficiency of siRNA silencing between cell-lines.” If they know this, why do they keep changing, instead of keeping one cell line and then comparing with another to validate their model? They should repeat all the critical experiments in one cell line and include the supportive data from other cell lines in the supplementary data. This is particularly important as the data supporting the title (ATG9 trafficking) are entirely based on experiments done in one clone of SNX4 KO HeLa cells, while most of the other data are done in RPE1 cells.
2. A similar problem is the use of different stimuli/chemicals to induce or inhibit autophagy in various experiments. To induce autophagy, they use either 1 hr starvation or 2 hrs inhibition of mTOR (AZD). To block autophagic flux they either use NH_4Cl or Bafilomycin 1. Again, a strength if one can confirm same phenotype with two parallel approaches, but suspicious if used for different read-outs.
3. It is also not clear why they use GFP-LC3B hTERT RPE1 cells in Fig. 1E and YFP-LC3B hTERT RPE1 cells in Fig. 1F. The two fluorochromes do have a slightly different emission peak.
4. The authors have used SMART-pool siRNA to deplete SNX4, SNX7 and/or SNX30, containing a mix of four single siRNA oligos. They should repeat the key experiments with two single oligos to confirm their data and reduce the risk of off-target effects (which are larger with SMARTpools). Such off-target effects could account for some of the differences seen, e.g. WIPI2 puncta numbers in hTERT-RPE1 cells silenced for SNX4 are significantly higher compared to control, but in SNX4 KO WIPI puncta don’t differ from wild-type. Why use two different control siRNAs that change between different experiments?
5. The authors make SNX4 KO HeLa cells (using CRISPR/Cas9) and show nicely that they can rescue LC3B puncta formation by transient expression of SNX4 in these cells. These cells are then used in Fig. 6 and 7. It is however not clear which KO clone is used for these experiments. In supplementary Fig. 5, they show several SNX4 KO clones immunoblotted for various proteins and there seems to be great clonal variation, even between clones that seem to lack SNX4 (e.g. SNX30 levels are greatly reduced in clone A, but similar to WT for clone C and D). The first figure doesn not show B, but the graph quantifies B…. They need to show critical experiments with more than

© 2020. Published by The Company of Biologists under the terms of the Creative Commons Attribution License (https://creativecommons.org/licenses/by/4.0/).

one KO clone or at least make a stable SNX4 rescue cell line and show that the SNX4 KO phenotypes can be rescued.
6. The authors claim that autophagy flux is reduced in cells lacking SNX4, mostly based on reduced LC3B lipidation and number of LC3B spots. It is then very surprising to see in Fig. 6D-E that the total number of mCherry-GFP-LC3B puncta and also the number of red only puncta are increased in SNX4 KO cells compared to control cells. How can this be explained? The authors should use other assays (e.g. degradation of long-lived proteins) to confirm that autophagic flux is indeed reduced in SNX4 depleted cells. The fed and starve p62 flux samples (Fig. 4C) should ideally be run on the same gel and all samples should be normalized to the fed no BafA1 condition to see p62 flux.
7. The authors show several blots where SNX4 KD leads to reduced levels of SNX7 and SNX30 (e.g. Fig 2C). They should do qPCR analysis to make sure this is a protein stability effect and not due to reduced transcription.
8. They say initially that they have done a siRNA-based LC3B lipidation screen of human SNXs 1-30. They should either show the data or explain why they choose not to and specify how this screen was done.
Especially as they based on this discuss differences with SNX18 throughout the text. It is a bit confusing that they initially state "….we found no evidence for involvement of any other SNXs during starvation-induced autophagy" (should specify any other than SNX4) and in the discussion state but "neither SNX4 nor SNX18 were identified as autophagy regulators in the respective, alternate siRNA-based screens (this study; (Knaevelsrud et al., 2013))".
9. The resolution of several images is not sufficient to see dots (e.g. Fig 1B and E, 4E,…)
10. The figure organisation is sometime confusing, particularly difficult to know which graph belongs to which panel (e.g. Fig. 1B graph). Thus, each graph should be labelled as an individual sub-figure.
11. MW standards should be indicated for all blots
12. Statistics; it is very nice that most of the imaging data have been quantified, but it is not so clear how the statistics have been done. They should indicate for each figure how many cells were quantified per condition from how many experiments and how the statistical significance was calculated. Each figure should indicate n= and if SD or SEM are used. In general, for puncta quantification they claim that "For a typical experiment fifteen random fields were imaged and puncta numbers per cell in each field was counted", which is fine. However, sometimes the size of the error bars are very large, but at the same time the significance value is very low, which makes me think that they might have used the individual fields as independent values. For calculation of significance, they should rather use the value of the average puncta number per experiment from >3 experiments. The same argument goes for the quantification of colocalization (state that 25 cells per condition used for quantification, but not how many experiments or how significance calculated). E.g. in Figure 5D, the error bars are very large, yet significant results.
13. It might be helpful with a model to summarize the main findings of their paper.

Specific comments:
1. The nomenclature of SNX4 and its homolog in yeast is somewhat confusing and should be unified. Examples; "SNX4 (also known as ATG24B and CVT13)", "Snx4/Atg24", "deletion of ATG24B or ATG20"…
2. Page 3. "Mammalian cells possess twelve SNX-BAR family members—SNX1, SNX2, SNX4 through to SNX9, SNX18, SNX30, SNX32 and SNX33" It would be convenient to write out all the proteins (especially as they work with SNX7).
3. The introduction and discussion could be shortened.

FIGURE 1:
1A: ATG5 KD efficiency should be shown. aLC3B (long) should be (long exposure).
1B: Why not do the quantification of LC3 spots +/- BafA1 (or NH_4Cl)??
1D: only one of the many mCherry-SNX4 spots colocalize with GFP-ATG5. How can they rule out overexpression artifacts?
1E: The starved cell seems to have a higher level of SNX4. Does expression of SNX4 increases during starvation or is degradation affected?
1F: in the text, authors claim to repeat the experiment in the absence or presence of Bafylomicin A1 but this in not reported in the figure.

FIGURE 2:
2A: The "key" labeling should be changed to better separate the upper and lower part.

© 2020. Published by The Company of Biologists under the terms of the Creative Commons Attribution License (https://creativecommons.org/licenses/by/4.0/).

FIGURE 3:
Fig.3 legend “The requirement for SNX4 coexpression is further assessed in Fig. S3” - should be Fig S4. In Fig S4 they titrate mCherry-SNX4 levels, but use FLAG-SNX4 in Figure 3. They should also show representative pictures of FLAG-SNX overlap with SNX7 and SNX30. The fact that they need to express FLAG-SNX4 to be able to see SNX7 and SNX30 seems a bit weird (Fig. 4S). How does this affect localization/function of SNX7/30 in cells where only SNX4 is overexpressed?

FIGURE 4:
- some graphs just show the starved bars (and others show control and starved) – they should compare puncta in starved cells to fed.
4A: Should also do co-KD of SNX7 + SNX30.
4E and H: There is a lot of GFP-ATG13 and -ATG5 in the cell nucleus and very difficult to see cytoplasmic puncta for GFP-ATG13 - how are the puncta quantified?
4F and H: Should indicate if the cells shown are starved or not.
4I: what is siGL2?
4I: why combinatory KD’s only for this figure?

FIGURE 5:
- they quantify colocalization between ATG5 and ATG16L1 (graph use yeast nomenclature) and between ATG5 and WIPI2 (WIPI2B?) and conclude that “SNX4/SNX7 depletion impacts on autophagy at the ATG5 recruitment/retention stage”. They should also quantify colocalization between ATG16L1 and WIPI2.

FIGURE 6:
6D. Have they chosen the correct pictures to represent their graph? E.g. AZD+BafA1 Control has a lot of red in the image they provide, and that does not match at all with the graph. They should set the intensities of the lasers so that there are only yellow dots seen in cells treated with BafA1 (given the BafA1 works) and then use the same settings for all pictures. Here they have some cells that are green, which should not happen as they look at mCherry-GFP-LC3B. See also comment above regarding SNX4 KO phenotype.

Figure 7:
7A: authors report a “strongly colocalisation” but from the image this rather seems to be a juxtaposition for most of the dots.
7B, it would be great if they could do a zoom for the merge (as in A).

Figures 6C-7A-7C: in these images there is a double condition for the control (full nutrient and treatment with AZD), but only one condition (AZD) for SNX4 KO, while all four conditions are reported for both in the graphs.
They should also include images for SNX4 KO fed.

Supplementary figures:
Figure S2A. They see that under SNX4 OE, EEA1 positive structures diminish, which is rather strange as GFP-SNX4 hardly colocalize with EEA1. Is endocytosis (e.g. EGFR degradation) affected by SNX4 overexpression?
Figure S2B: The authors claim “Analysis of CD63-positive lysosomes revealed significantly more lysosomes in fed GFP-SNX4 expressing hTERT-RPE1 cells, but a clear absence of induced CD63 puncta increases following amino acid/growth factor starvation” How can they claim that those are lysosomes as CD63 can be either on the lysosome or in the endosomes? They should confirm with e.g. lysotracker or Lamp staining.
In both cased (A and B) it is not clear what these data indicate. Does SNX4 KD also regulate endosome/lysosome numbers?

Reviewer 2

Advance summary and potential significance to field

In the manuscript entitled “A heterodimeric SNX4:SNX7 SNX-BAR autophagy complex coordinates ATG9A trafficking for efficient autophagosome assembly” by Anton et al., the authors describe that

© 2020. Published by The Company of Biologists under the terms of the Creative Commons Attribution License (https://creativecommons.org/licenses/by/4.0/).

SNX4 is important for autophagosome formation in mammalian cells. They also showed that SNX4 forms heterodimers with either SNX7 or SNX30. Live imaging analysis shows that the lifespan of ATG5 puncta in SNX4-knockdown cells is shorter than that in wild-type cells. Finally, they show the intracellular dynamics of ATG9A alters in SNX4-knockout cells. These findings are interesting, supported by the convincing data. I find the manuscript to be potentially worthy of publication in Journal of Cell Science, but some concerns are remaining.

Comments for the author

[Major concern]
1) Figures 5 and 7: From the results shown in Figures 5 and 7, the authors conclude that “ATG5 recruitment and/or turnover kinetics are altered in SNX4-suppressed cells” and “Together, these data suggest that SNX4 contributes to the steady state localization of ATG9A, and that SNX4 is required for efficient ATG9A peripheral redistribution upon autophagy stimulation to enable efficient autophagy responses”. I do not feel that these phenomena are independent. I recommend the author to examine the dynamics of ATG5 in ATG9A-knockout/knockdown cells as described in Figure 5D–F although they mentioned a previous report that describes ATG16 puncta decrease in ATG9A-knockout cells (Orsi et al., 2012) in the discussion section. This experiment will clarify the functional relationship between ATG5 and ATG9A.

[Specific comments]
2) Page 7, line 28: Please cite a paper that describes AZD8055 as an inhibitor of mTORC1/2.
3) Page 7, line 32: There are two ‘further’ in the sentence.
4) Figure 1B: The reviewer cannot recognize LC3 puncta in starved control cells.
I recommend to modify the images to clearly visualize LC3 puncta using grayscale images. Moreover, it is difficult to recognize the characters “DAPI” in color images. Please modify these problems.
5) Figure 1D: It is difficult for the reviewer to recognize the labelling “CFP-LC3B” in the color image. Also, localization of CFP-LC3B is difficult to recognize in the image. The reviewer recommends to show grayscale images of individual colors.
6) Legend of Figure 1E: The expression of “Example fields to the left; quantitation to the right.” is probably incorrect. “Example fields to the lower; quantitation to the upper.” might be right. The reviewer recommends to rearrange the panels.
7) Figures 4 and 6: It is hard to recognize the labelling “DAPI” in color images.

First revision

<u>Author response to reviewers' comments</u>

This rebuttal was formulated with advice from the Editor and the Editorial Office with consideration of laboratory closures due to the COVID emergency and an inability to conduct experiments. We have included additional data where we had these available.

Reviewer 1 Advance Summary and Potential Significance to Field:

The manuscript by Anton et al. entitled “A heterodimeric SNX4:SNX7 SNX-BAR autophagy complex coordinates ATG9A trafficking for efficient autophagosome assembly” reports on a role for heterodimer SNX4:SNX7 in the autophagosome formation. The authors confirm that SNX4 is a core component of two heterodimers (SNX4:SN7 and SNX4:SNX30) (as previously shown (Van Weering et al., 2012).

The concept of mammalian SNX4:SNX7 and SNX4:SNX30 heterodimers was originally proposed by our group back in 2012. As we state in the text, these earlier experiments were carried out using overexpressed, tagged proteins. The advance here is that we confirm partner interactions at the level of endogenous proteins, and importantly, we also show that the stability of the partner (i.e. SNX7/SNX30) is governed by the presence of the core SNX4 component. These new data are important

© 2020. Published by The Company of Biologists under the terms of the Creative Commons Attribution License (https://creativecommons.org/licenses/by/4.0/).

for the narrative of the paper, in which we identify SNX7 as the second component of the SNX4 autophagy heterodimer.

They present further evidence that both complexes localize to early endosomes and that depletion of SNX4 prevents efficient lipidation of LC3B and leads to a loss of LC3 puncta both in fed and in starved conditions. To elucidate the role of the SNX4 complexes in autophagy, they do a series of siRNA experiments to analyze how depletion of the different components affects numbers and localization of early and late autophagy markers and conclude that the SNX4:SNX7 complex regulates ATG9 trafficking. This is a potentially interesting study as it represents a novel role for the heterodimer SNX4:SNX7 in autophagy. However, the data presented in the manuscript must be strengthened to support the conclusions made, as indicated below.

Reviewer 1 Comments for the Author:
General comments:

1. The authors use several different cell lines throughout the manuscript, which in principle is fine (and a strength) as long as the results obtained in one cell line is confirmed in another cell line.

For the siRNA experiments, we presented data using both RPE1 cells (various Figs) and GFP-LC3B HEK293 cells (Fig. 4A), and went on to provide focused supporting data from experiments using SNX4 CRISPR null HeLa cells. It is not common practice to repeat biochemical (e.g. pull-down experiments) and CRISPR KO experiments in different cell lines, and we feel that this is an unreasonable suggestion. If the referee is instead suggesting that we confirm some of the more mechanistic observations (e.g. differences in ATG5 kinetics) in a different cell-line, we strongly feel that this goes beyond what is normally expected for a cell biology study such as this. The overarching take-home of this study is that suppression of SNX4 causes a reduced autophagy response, and we show this in 3 human cell-lines. Overall then, we believe that the sum and clarity of our extensive data ought to be sufficient for publication in the Journal of Cell Science.

Here however, it seems like the authors have done different experiments in different cell lines, which could give the impression that one has cherry-picked results to make a story. They have used hTERT-immortalized retinal pigment epithelial (hTERT-RPE1) cells for most of the initial autophagy experiments, but then use HeLa cells to IP the SNX4 complexes and generate a CRISPR-Cas9 knock out of SNX4 and also HEK293 GFP-LC3 cells.

We use hTERT-RPE1 cells for much of our mechanistic autophagy research because these cells are not transformed, have low basal autophagy rates, respond well to autophagy stimuli, and are configured for debris clearance and tissue homeostasis in the oxidative environment in the eye. This makes these excellent/robust autophagy models. They are, however, less suitable for IP experiments and general biochemistry because they do not transfect as easily as e.g. HeLa or HEK293 cells. Whilst it is possible to make CRISPR knockouts in the RPE1 background (e.g. Stevenson et al. 2017 Journal of Cell Science 130: 4132-4143), efficiency of Cas effector transfection and cell growth properties (e.g. lack of colony formation; cell migration) mean that CRISPR work in these cells is extremely challenging. For this reason, we opted to use HeLa cells, because of their suitability for CRISPR work.

They state “This probably reflects cell-type specific differences in the relative balance of SNX4-containing heterodimers and/or the efficiency of siRNA silencing between cell-lines.” If they know this, why do they keep changing, instead of keeping one cell line and then comparing with another to validate their model?

It is not accurate to suggest that we “keep changing” cell-lines. The siRNA-based autophagy experiments are all carried out in RPE1 cells, with one validation LC3 puncta count experiment carried out in HEK293s.
They should repeat all the critical experiments in one cell line and include the supportive data from other cell lines in the supplementary data.
This is an unreasonable demand. For consistency, we have moved the HEK293 data (formerly Fig. 4A) into the supplemental section (New Fig. S1B), so that the autophagy siRNA experiments shown in the main text are based in RPE1 cells.

© 2020. Published by The Company of Biologists under the terms of the Creative Commons Attribution License (https://creativecommons.org/licenses/by/4.0/).

This is particularly important as the data supporting the title (ATG9 trafficking) are entirely based on experiments done in one clone of SNX4 KO HeLa cells, while most of the other data are done in RPE1 cells.
It is extremely rare to see experiments repeated using several CRISPR clones in cell biology journal such as JCS. In this study, we have included rescue experiments in our characterised SNX4 KO clone (Fig. 6G; 7B), which goes beyond that which is commonly encountered in similar studies. We also now provide additional data in Fig. S6C (shown below as Rebuttal Fig. 2) further LC3B and WIPI2 puncta data analysis in a second CRISPR clone.

2. A similar problem is the use of different stimuli/chemicals to induce or inhibit autophagy in various experiments. To induce autophagy, they use either 1 hr starvation or 2 hrs inhibition of mTOR (AZD). To block autophagic flux they either use NH4Cl or Bafilomycin 1. Again, a strength if one can confirm same phenotype with two parallel approaches, but suspicious if used for different read-outs.
We regret that the referee appears suspicious of our work. For consistency, we have used amino acid/growth factor starvation for the majority of our studies, and use AZD only for the HeLa SNX4 CRISPR null (excepting one additional AZD dataset shown in Fig. S4). This is because the HeLa sub-type used for the CRISPR work does not respond robustly to amino acid/growth factor withdrawal. When we began this study some years ago we were routinely using NH_4Cl for lysosomal inhibition; however, more recently, we have switched to BafA1. We believe that this is very minor issue that does not in any way affect the interpretation of the data, and regret that the referee has chosen to find problems with the data, rather than focus on critical advice to improve the manuscript.

3. It is also not clear why they use GFP-LC3B hTERT RPE1 cells in Fig. 1E and YFP-LC3B hTERT RPE1 cells in Fig. 1F. The two fluorochromes do have a slightly different emission peak.
We are grateful that the referee noted this. This was a labeling error; the cells in Fig. 1E were YFP-LC3B RPE1 cells. The different emission peaks of the 2 fluorophores would in any case not have affected the interpretation of the data.

4. The authors have used SMART-pool siRNA to deplete SNX4, SNX7 and/or SNX30, containing a mix of four single siRNA oligos. They should repeat the key experiments with two single oligos to confirm their data and reduce the risk of off-target effects (which are larger with SMARTpools).

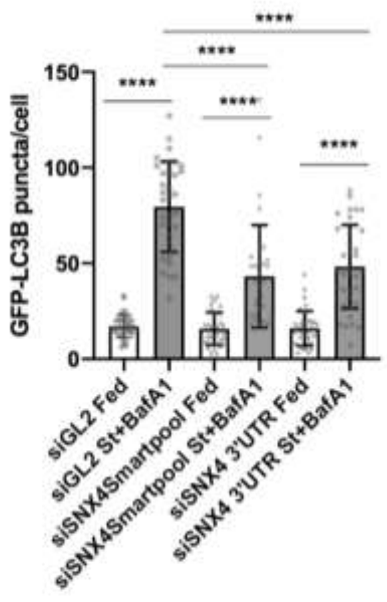

The referee is mistaken on this point; SMARTpools are designed to reduce off-target effects. Whilst increasing the theoretical potential (x4) to "hit" off-target transcripts, the relative concentrations of each individual oligonucleotide in the pool is reduced to 25%; a concentration that is unlikely to result in substantial transcript silencing even with high-fidelity targeting. The request to carry out supporting experiments with individual oligos is not an unreasonable one (although 2 additional oligos would be beyond what is commonly encountered); however, we do not agree with the practice of selecting individual oligos from the original SMARTpool, as this exposes researchers to increased off-target silencing by ramping up the effective concentrations of the single oligos.
We now include partial data (fed; starved+BafA1) that we had available using one additional siRNA oligo targeting the 3'UTR, and these data are shown in Rebuttal, Fig. 1. This pilot experiment was performed in view of siRNA/rescue experiments, but we opted for CRISPR route with rescue data. We argue that this ought to be sufficient this study, and now include these data as part of an updated supplementary figure (Fig. S1A).
Rebuttal Fig. 1: GFP-LC3B puncta in stable hTERT-RPE1 cells transfected with siControl, siSNX4 SMARTpool, and siSNX4 3'UTR. Data show basal (fed) and starved+BafA1 (1hr).

Such off-target effects could account for some of the differences seen, e.g. WIPI2 puncta numbers in hTERT-RPE1 cells silenced for SNX4 are significantly higher compared to control, but in SNX4 KO WIPI puncta don't differ from wild-type.

© 2020. Published by The Company of Biologists under the terms of the Creative Commons Attribution License (https://creativecommons.org/licenses/by/4.0/).

We feel that it is much more likely this is due to the differences between acute (siRNA) and chronic (CRSIPR KO) SNX4 loss and the functional compensation that is necessary for CRISPR null cells to thrive in the absence of a selected gene product. The likelihood that the autophagy impairments recorded following siRNA SMARTpool transfection being due to an off-target affect, with CRISPR editing of the same target also causing the same pathway defect (i.e. autophagy) would seem to be quite small.

Why use two different control siRNAs that change between different experiments?

The initial SNX screening studies were carried out in the Cullen lab using siControl 1; the mechanistic autophagy studies were carried out in the Lane lab using siControl 2. To be entirely open, we have chosen to list the sequences of both siControls, and to show these difference in the specific figures; however, we feel that this is probably adding to confusion, so instead propose using "siControl" in the body of the text, whilst indicating in the Materials and Methods those figure parts (Fig. 1A; 4A) where a different control siRNA was used.

5. The authors make SNX4 KO HeLa cells (using CRISPR/Cas9) and show nicely that they can rescue LC3B puncta formation by transient expression of SNX4 in these cells. These cells are then used in Fig. 6 and 7. It is however not clear which KO clone is used for these experiments.

We stated clearly on page 13: "We selected clone "A" for detailed analysis as these cells showed depleted SNX7 and SNX30 alongside an absence of SNX4".

In supplementary Fig. 5, they show several SNX4 KO clones immunoblotted for various proteins and there seems to be great clonal variation, even between clones that seem to lack SNX4 (e.g. SNX30 levels are greatly reduced in clone A, but similar to WT for clone C and D). The first figure doesn not show B, but the graph quantifies B…. They need to show critical experiments with more than one KO clone or at least make a stable SNX4 rescue cell line and show that the SNX4 KO phenotypes can be rescued.

The layout of Fig. S5 may have confused the referee - the immunoblots to the left are representative blots from an extensive characterization of multiple clones; to the right is the quantitation. The second blot on the left does contain the data for clone B. We have rearranged, and split this figure up into parts for clarity. Note that we chose to show the data from multiple clones in the supplemental information because we feel it is important to emphasise how different CRISPR clones compensate in different ways for the loss of a particular protein. We are in somewhat a privileged position because the clear relationships between SNX4 and the stability of SNX7/30 enables us to record directly differences between clones, and this is the reason we wanted to show the characterization data to a level that is usually absent in papers.

We think that showing critical experiments with more than one clone would be a reasonable request (under normal circumstances), but it is certainly not common practice within the cell biology field to see multiple CRISPR clones and/or stable knockdown data presented as a matter of course. Thus, this referee is demanding a level of rigour that is simply not encountered in the field, and we wonder why this might be? We believe that given the large amount of data contained in this paper, the rescue data in Fig. 6G ought to be sufficient for the Journal of Cell Science. We have, however, collected full LC3/WIPI2 puncta data for a second clone (Clone B; in which SNX7 and SNX30 proteins levels are also reduced following SNX4 KO), and these data are shown below (Rebuttal Fig. 2). We have opted to show these data alongside the clonal characterization as new Fig. S6C.

© 2020. Published by The Company of Biologists under the terms of the Creative Commons Attribution License (https://creativecommons.org/licenses/by/4.0/).

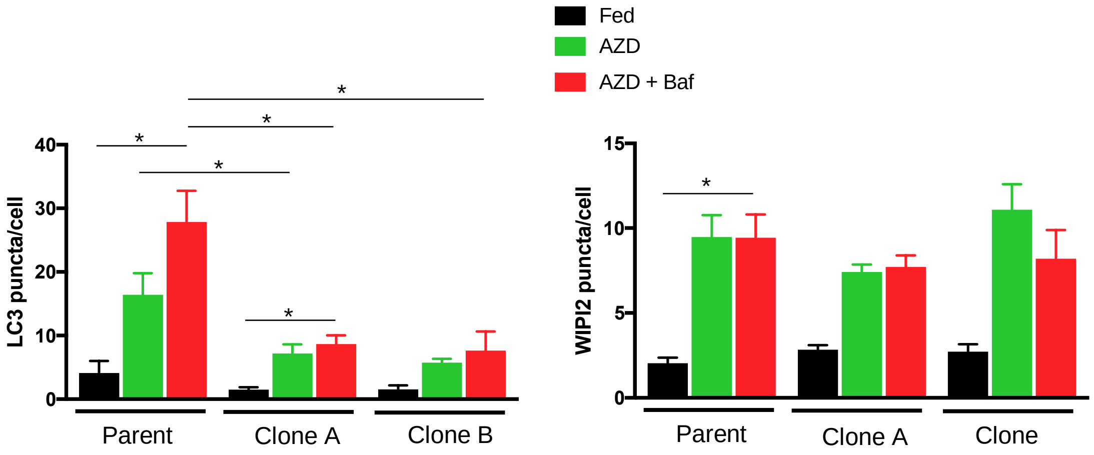

Rebuttal Fig. 2: LC3B and WIPI2 puncta counts for 2 SNX4 CRISPR KO clones. Clone A is used in the paper; Clone B is provided for comparison.

6. The authors claim that autophagy flux is reduced in cells lacking SNX4, mostly based on reduced LC3B lipidation and number of LC3B spots. It is then very surprising to see in Fig. 6D-E that the total number of mCherry-GFP-LC3B puncta and also the number of red only puncta are increased in SNX4 KO cells compared to control cells. How can this be explained?

We were also not expecting to see this. It is clear, however, that at basal, steady state, mCherry-positive puncta are more numerous in the SNX4 KO cells. This is why the total numbers of puncta are higher. We think this is probably due to different rates of mCherry lysosomal degradation in parental and SNX4 KO cells. Since our basic analysis by CD63+ve puncta counting suggests differences in the late endosomal/lysosomal compartment in SNX4 siRNA depleted cells (Fig. S3B), we would argue that in the absence of SNX4, lysosomal function may be perturbed. We intend to follow this up in future work. Note that our detailed analysis of autophagy in SNX4 depleted cells suggests impairments at the autophagosome assembly stage, not at the level of flux.

The authors should use other assays (e.g. degradation of long-lived proteins) to confirm that autophagic flux is indeed reduced in SNX4 depleted cells. The fed and starve p62 flux samples (Fig. 4C) should ideally be run on the same gel and all samples should be normalized to the fed no BafA1 condition to see p62 flux.

The data in this figure were normalized against control siControl/-BafA1 values within treatment groups, and we apologise for not clarifying this. This is now indicated in the figure legend. The referee is correct to note that we ought to have run these on the same gel to allow assessment of p62 degradation as a function of starvation. The experiment was not carried out that way because the samples exceeded space on a standard 15-well mini gel. As these are normalized against GAPDH and against siControl/-BafA1 within their own treatment groups, the data do report differences between siRNA conditions, so are informative in that sense.

We have carried out p62 degradation assays in which all relevant data are on the same gel, and example is shown in Rebuttal Fig. 3. These were set up using long starvation/BafA1 treatments (4hrs/6hrs; n=2) and showed very little evidence of flux differences. Parallel analysis (n=1) using AZD was more promising; however, we opted to repeat these experiments in the starvation setting for the paper, using shorter treatments, and including BAfA1 only data (Fig. 4C in the main submitted text).

Degradation of long-lived protein assays are not straightforward, are not common practice in the field. Our lab is not set up to do this (even when we re-open post COVID), and we feel would be an unreasonable suggestion for the current study.

“NOTE: We have removed unpublished data that had been provided for the referees in confidence.”

© 2020. Published by The Company of Biologists under the terms of the Creative Commons Attribution License (https://creativecommons.org/licenses/by/4.0/).

7. The authors show several blots where SNX4 KD leads to reduced levels of SNX7 and SNX30 (e.g. Fig, 2C). They should do qPCR analysis to make sure this is a protein stability effect and not due to reduced transcription.

Under normal circumstances, this would not an unreasonable suggestion, although the likelihood of their being epigenetic silencing of expression of the other genes is highly unlikely. We suggest amending the sentence on page 9 to read: "Although changes at the transcriptional level cannot be excluded, we propose that upon SNX4 suppression, the loss of the core component destabilises the other constituents, while upon suppression of an individual complex specific component, such as SNX7, the presence of the core SNX4 component allows the stabilisation of the SNX4:SNX30 complex (and vice versa under conditions of SNX30 suppression)." (added text is underlined).

8. They say initially that they have done a siRNA-based LC3B lipidation screen of human SNXs 1-30. They should either show the data or explain why they choose not to and specify how this screen was done. Especially as they based on this discuss differences with SNX18 throughout the text. It is a bit confusing that they initially state "….we found no evidence for involvement of any other SNXs during starvation-induced autophagy" (should specify any other than SNX4) and in the discussion state but "neither SNX4 nor SNX18 were identified as autophagy regulators in the respective, alternate siRNA-based screens (this study;(Knaevelsrud et al., 2013))".

"NOTE: We have removed unpublished data that had been provided for the referees in confidence."

We agree that mentioning this while not showing the data is, in hindsight, confusing/unhelpful, but were not comfortable with showing the data because of its very preliminary nature. This is partly because while obtaining funding for the SNX4 work, and while in the process of advancing the study, the original Knævelsrud et al. 2013 paper was published in JCB. Rather than work to repeat a full screen (as this was carried out in the Knævelsrud study), we chose instead to focus on SNX4 biology as there was existing strong evidence for a role for this protein in autophagy in yeast. We therefore suggest removing mention of the screen in the manuscript, page 6 (Introduction), i.e.: "In siRNA-based LC3B lipidation screens of human SNXs 1-30, we found no evidence for involvement of any other SNXs during starvation-induced autophagy (three independent tests in hTERT-RPE1 cells; data not shown). This panel included SNX18, a SNX-BAR previously shown to regulate autophagy in an imaging-based autophagy screen (Knaevelsrud et al., 2013). The lack of an effect on LC3 lipidation following treatment with SNX18 siRNA in our hands can probably be attributed to cell-type differences, assay sensitivity (imaging vs. immunoblotting), and/or siRNA efficiency."

9. The resolution of several images is not sufficient to see dots (e.g. Fig 1B and E, 4E,…)

We have improved the size/clarity of the images in all relevant figures throughout, including most notably, providing inverted greyscale puncta/label overlays on DAPI (blue), and feel that this has worked very well. It is not appropriate to change the resolution without resampling the images.

10. The figure organisation is sometime confusing, particularly difficult to know which graph belongs to which panel (e.g. Fig. 1B graph). Thus, each graph should be labelled as an individual sub-figure.

We thank the referee for noting this. We have reorganized/relabeled the figures.

11. MW standards should be indicated for all blots

We apologise for the MW standards that were missing in Figs 1A and 2C. These have now been added.

12. Statistics; it is very nice that most of the imaging data have been quantified, but it is not so clear how the statistics have been done. They should indicate for each figure how many cells were quantified per condition, from how many experiments and how the statistical significance was calculated. Each figure should indicate n= and if SD or SEM are used. In general, for puncta quantification they claim that "For a typical experiment, fifteen random fields were imaged and puncta numbers per cell in each field was counted", which is fine. However, sometimes the size of the error bars are very large, but at the same time the significance value is very low, which makes me think that they might have used the individual fields as independent values. For calculation of significance, they should rather use the value of the average puncta number per experiment from >3

© 2020. Published by The Company of Biologists under the terms of the Creative Commons Attribution License (https://creativecommons.org/licenses/by/4.0/).

experiments. The same argument goes for the quantification of colocalization (state that 25 cells per condition used for quantification, but not how many experiments or how significance calculated). E.g. in Figure 5D, the error bars are very large, yet significant results.

We apologise for not being clearer about the way in which experiments were sampled and statistics applied. We have sought to clarify each of these with statements in the respective figure legends. We have also included individual data points on most of the graphs. We did not use individual fields as independent values

13. It might be helpful with a model to summarize the main findings of their paper.

At the request of the reviewer, we have included a summary model in Fig. 7E.

Specific comments:

1. The nomenclature of SNX4 and its homolog in yeast is somewhat confusing and should be unified. Examples; "SNX4 (also known as ATG24B and CVT13)", "Snx4/Atg24", "deletion of ATG24B or ATG20"…

To our knowledge we have complied with the standard for species-specific gene and protein nomenclature, and have included alternative yeast names for clarity. We do not feel that it is our role to unify the nomenclature; however, we have tried to improve clarity where possible (e.g. page 4: "… yeast Atg11 assembles with Snx4/Atg24 and Atg20, replacing the non-selective autophagy Atg1 sub-complex", we have changed to simply read "…Snx4 and Atg20…").

2. Page 3. "Mammalian cells possess twelve SNX-BAR family members—SNX1, SNX2, SNX4 through to SNX9, SNX18, SNX30, SNX32 and SNX33" It would be convenient to write out all the proteins (especially as they work with SNX7).

We do not feel that it is necessary to change this.

3. The introduction and discussion could be shortened.

We thank the referee for this observation, but feel that whilst we remain within the character limit, this study needs the level of narrative that we have provided.

FIGURE 1:

1A: ATG5 KD efficiency should be shown. aLC3B (long) should be (long exposure).

We have clarified this in the legend. We do not have ATG5 blots available; however, these siRNAs were first used in Betin et al. (2009) JCS 122: 2554-2566, and example immunoblots can be found here.

1B: Why not do the quantification of LC3 spots +/- BafA1 (or NH4Cl)??

Because the flux analysis comes later. We feel that describing the data in this order makes the narrative clearer.

1D: only one of the many mCherry-SNX4 spots colocalize with GFP-ATG5. How can they rule out overexpression artifacts?

We cannot rule this out. This is simply a qualitative observation carried out using transiently overexpressed proteins. In RPE1 cells there are very few ATG5 puncta at any given time (see MacVicar et al. (2015) International Journal of Molecular Sciences 16: 13356-13380).

1E: The starved cell seems to have a higher level of SNX4. Does expression of SNX4 increases during starvation or is degradation affected?

This is simply a consequence of transient expression giving differing expression levels between cells.

© 2020. Published by The Company of Biologists under the terms of the Creative Commons Attribution License (https://creativecommons.org/licenses/by/4.0/).

1F: in the text, authors claim to repeat the experiment in the absence or presence of Bafylomicin A1 but this in not reported in the figure.

We have amended this sentence to read "…and this pattern was repeated following starvation in the presence of the vacuolar…"

FIGURE 2:
2A: The "key" labeling should be changed to better separate the upper and lower part.

We have altered this to make the figure clearer.

FIGURE 3:

Fig.3 legend "The requirement for SNX4 coexpression is further assessed in Fig. S3" - should be Fig S4.

This has now been corrected.

In Fig S4 they titrate mCherry-SNX4 levels, but use FLAG-SNX4 in Figure 3. They should also show representative pictures of FLAG-SNX overlap with SNX7 and SNX30.

We are not convinced that this would add much to the data.

The fact that they need to express FLAG-SNX4 to be able to see SNX7 and SNX30 seems a bit weird (Fig. 4S). How does this affect localization/function of SNX7/30 in cells where only SNX4 is overexpressed?

We did not understand this comment, and feel that a study looking at the functions of SNX7/30 falls beyond the scope of the current work.

FIGURE 4:

-some graphs just show the starved bars (and others show control and starved) – they should compare puncta in starved cells to fed.

Using anti-ULK1 and anti-ATG16L1 antibodies (these are not the best for immunofluorescence, and the latter also stains the centrosome) and the stable ATG13-GFP cell-line, puncta numbers in the fed state are very low (typically <1 per cell), meaning that readouts are not particularly reliable. This is why for these markers we are showing the starved data only.
For clarity, we have amended Fig. 4 by showing consistent data formats between the different markers where possible, and removing the WIPI2 double suppression data to the Supplemental section (Fig. S1C).

4A: Should also do co-KD of SNX7 + SNX30.

We feel that this is just suggesting experiments for the sake of it. There is one example of this shown in Fig. S1C.

4E and H: There is a lot of GFP-ATG13 and -ATG5 in the cell nucleus and very difficult to see cytoplasmic puncta for GFP-ATG13 - how are the puncta quantified?

Puncta are automatically quantitated using software in MetaMorph. Multiple fields of cells are imaged, TopHat algorithm for object size/shape and thresholded, before the computer automatically counts puncta as a function of cell number. Nuclear fluorescence is not unusual with GFP fusions, and tends to be fusion construct size-dependent. We have re-coloured the images for the sake of clarity.

4F and H: Should indicate if the cells shown are starved or not.

This has now been added.

© 2020. Published by The Company of Biologists under the terms of the Creative Commons Attribution License (https://creativecommons.org/licenses/by/4.0/).

4I: what is siGL2?

Apologies. This was labelled wrongly- it should have read "siControl no. 2". But see note above on proposed siControl nomenclature changes.

4I: why combinatory KD's only for this figure?

Simply to provide an example of how combinatorial suppression affects puncta formation at the level of an endogenous marker. Combinatorial data have now been removed, with a single example placed in Fig. S1C.

FIGURE 5:-they quantify colocalization between ATG5 and ATG16L1 (graph use yeast nomenclature) and between ATG5 and WIPI2 (WIPI2B?) and conclude that "SNX4/SNX7 depletion impacts on autophagy at the ATG5 recruitment/retention stage". They should also quantify colocalization between ATG16L1 and WIPI2.

This is a reasonable suggestion, and data are now included in the Figure. There are no significant differences, which is perhaps surprising; however, this is probably due to the high levels of cytoplasmic background using these markers.

FIGURE 6:

6D. Have they chosen the correct pictures to represent their graph? E.g. AZD+BafA1 Control has a lot of red in the image they provide, and that does not match at all with the graph. They should set the intensities of the lasers so that there are only yellow dots seen in cells treated with BafA1 (given the BafA1 works) and then use the same settings for all pictures. Here they have some cells that are green, which should not happen as they look at mCherry-GFP-LC3B. See also comment above regarding SNX4 KO phenotype.

We appreciate the advice of the referee. Of course, we set our acquisition settings on the brightest/most abundant condition (starve/BafA1) and applied the same settings throughout. If settings are taken so that the cytoplasm is "yellow", as the referee is suggesting, then the bright lysosomal mCherry-LC3B signal becomes saturated. This is an issue that is very often overlooked when using tandem-tag LC3 constructs. The solution that we have applied (again as suggested by the referee, and one that is used throughout the field) is to gate for the brighter red, juxtanuclear autolysosomal objects. This has the inevitable consequence of an under-representation of peripheral, less bright red (early) autophagic puncta (and also cytosolic background). Although a compromise, this approach works quite well because the "yellow" puncta that are scored are effectively those that return a signal in the green channel. We have nevertheless adjusted the relative contrast/brightness of the channels for image clarity.

Figure 7: 7A: authors report a "strongly colocalisation" but from the image this rather seems to be a juxtaposition for most of the dots.

We have clarified these statements in the text.

7B, it would be great if they could do a zoom for the merge (as in A).

We appreciate this suggestion and have done as requested.

Figures 6C-7A-7C: in these images there is a double condition for the control (full nutrient and treatment with AZD), but only one condition (AZD) for SNX4 KO, while all four conditions are reported for both in the graphs. They should also include images for SNX4 KO fed.

We have omitted this simply for space and layout. As the data are fully quantitated, we are not convinced by the need to show these images.

© 2020. Published by The Company of Biologists under the terms of the Creative Commons Attribution License (https://creativecommons.org/licenses/by/4.0/).

Supplementary figures:

Figure S2A. They see that under SNX4 OE, EEA1 positive structures diminish, which is rather strange as GFP-SNX4 hardly colocalize with EEA1. Is endocytosis (e.g. EGFR degradation) affected by SNX4 overexpression?

We have not done this experiment and feel that, whilst an interesting question, this falls outside the remit of the current study.

Figure S2B: The authors claim “Analysis of CD63-positive lysosomes revealed significantly more lysosomes in fed GFP-SNX4 expressing hTERT-RPE1 cells, but a clear absence of induced CD63 puncta increases following amino acid/growth factor starvation” How can they claim that those are lysosomes as CD63 can be either on the lysosome or in the endosomes? They should confirm with e.g. lysotracker or Lamp staining. In both cased (A and B) it is not clear what these data indicate. Does SNX4 KD also regulate endosome/lysosome numbers?

This is a fair criticism. We have clarified this by stated that CD63 labels late endosomes and lysosomes. These are straightforward experiments to carry out, if the laboratory was open. We suggest that in SNX4 KD conditions, there are likely to be differences in the endocytic pathway, and this may be what we are recording here. That there are no changes following starvation is interesting, and this is something we would like to explore in future work. Given that the observed autophagy defect appears to be at the level of assembly (rather than flux), differences at the level of the late endosome/lysosome affecting autophagy are probably masked. Interestingly, we do see indirect evidence of inefficiencies in lysosomal function in the SNX4 CRISPR KO line, as discussed below.

Reviewer 2 Advance Summary and Potential Significance to Field:

In the manuscript entitled “A heterodimeric SNX4:SNX7 SNX-BAR autophagy complex coordinates ATG9A trafficking for efficient autophagosome assembly” by Anton et al., the authors describe that SNX4 is important for autophagosome formation in mammalian cells. They also showed that SNX4 forms heterodimers with either SNX7 or SNX30. Live imaging analysis shows that the lifespan of ATG5 puncta in SNX4-knockdown cells is shorter than that in wild-type cells. Finally, they show the intracellular dynamics of ATG9A alters in SNX4-knockout cells. These findings are interesting, supported by the convincing data. I find the manuscript to be potentially worthy of publication in Journal of Cell Science, but some concerns are remaining.

We are grateful that the referee sees merit in our work, and appreciate their input.

Reviewer 2 Comments for the Author:

[Major concern]

Figures 5 and 7: From the results shown in Figures 5 and 7, the authors conclude that “ATG5 recruitment and/or turnover kinetics are altered in SNX4-suppressed cells” and “Together, these data suggest that SNX4 contributes to the steady state localization of ATG9A, and that SNX4 is required for efficient ATG9A peripheral redistribution upon autophagy stimulation to enable efficient autophagy responses”. I do not feel that these phenomena are independent. I recommend the author to examine the dynamics of ATG5 in ATG9A-knockout/knockdown cells as described in Figure 5D–F although they mentioned a previous report that describes ATG16 puncta decrease in ATG9A-knockout cells (Orsi et al., 2012) in the discussion section. This experiment will clarify the functional relationship between ATG5 and ATG9A.

The referee describes an interesting set of experiments; however, we feel very strongly that these lie outside of the scope of the current study. Our approach (SNX4 KD/KO) does not markedly affect ATG9A stability - which is what a knockout/knockdown would do - so it is unclear how the data on ATG5 kinetics in an ATG9A depleted background would inform understanding of the role of SNX4/SNX7 in ATG9A trafficking. For the referee, we provide data showing that basal ATG9A levels are somewhat lower than in the parental line (significant for clone B), but that this does not appear

© 2020. Published by The Company of Biologists under the terms of the Creative Commons Attribution License (https://creativecommons.org/licenses/by/4.0/).

to be caused by segregation into different compartments. Thus, attempting to phenocopy this by ATG9 siRNA or KO would, we feel, be a different experiment.

“NOTE: We have removed unpublished data that had been provided for the referees in confidence.”

[Specific comments]

2) Page 7, line 28: Please cite a paper that describes AZD8055 as an inhibitor of mTORC1/2.

We have cited Chresta et al (2010) “AZD8055 Is a Potent, Selective, and Orally Bioavailable ATP-Competitive Mammalian Target of Rapamycin Kinase Inhibitor with In vitro and In vivo Antitumor Activity” Cancer Research 70: 288-298.

3) Page 7, line 32: There are two ‘further’ in the sentence.

This has been corrected.

4) Figure 1B: The reviewer cannot recognize LC3 puncta in starved control cells. I recommend to modify the images to clearly visualize LC3 puncta using grayscale images. Moreover, it is difficult to recognize the characters “DAPI” in color images. Please modify these problems.

These issues have been corrected by providing inverted greyscale images for markers overlaid on blue DAPI.

5) Figure 1D: It is difficult for the reviewer to recognize the labelling “CFP-LC3B” in the color image. Also, localization of CFP-LC3B is difficult to recognize in the image. The reviewer recommends to show grayscale images of individual colors.

These issues have been corrected

6) Legend of Figure 1E: The expression of “Example fields to the left; quantitation to the right.” is probably incorrect. “Example fields to the lower; quantitation to the upper.” might be right. The reviewer recommends to rearrange the panels.

This error has been corrected by text alterations in the figure legends.

7) Figures 4 and 6: It is hard to recognize the labelling “DAPI” in color images.

These issues have been corrected (see above).

Second decision letter

MS ID#: JOCES/2020/246306

MS TITLE: A heterodimeric SNX4:SNX7 SNX-BAR autophagy complex coordinates ATG9A trafficking for efficient autophagosome assembly

AUTHORS: Dr Zurine Anton, Virginie Betin, Boris Simonetti, Colin Traer, Naomi Attar, Peter Cullen, and Jon Lane
ARTICLE TYPE: Research Article

I have read your rebuttal carefully. Considering laboratory closure due to COVID-19 emergency and the validity of your rebuttal, I decided to accept your revised manuscript for publication in Journal of Cell Science, pending standard ethics checks.

© 2020. Published by The Company of Biologists under the terms of the Creative Commons Attribution License (https://creativecommons.org/licenses/by/4.0/).
